# SerpinA3k Deficiency Ameliorates Experimental type 2 Diabetes

**DOI:** 10.1007/s00018-025-05922-3

**Published:** 2025-12-02

**Authors:** Isaac González-Soria, Esmeralda Palacios-Brito, Dulce Gómez-Trujillo, Karla Barragán-Jiménez, Axel D. Soto-Valadez, Miguel Angel Martínez-Rojas, Daniel Oliva-García, Jorge Aranda, Rosalba Pérez-Villalva, Lorena López-Griego, Andrea Díaz-Villaseñor, Norma A. Bobadilla

**Affiliations:** 1 Universidad Nacional Autónoma de México, Mexico City, Mexico; 2 Instituto Nacional de Ciencias Médicas y Nutrición Salvador Zubirán, Mexico City, Mexico; 3 (PECEM) Program, Facultad de Medicina, Universidad Nacional Autónoma de México, Mexico City, Mexico; 4 Facultad de Química, Universidad Nacional Autónoma de México, Mexico City, Mexico

**Keywords:** Obesity, Adipocytes, Renal dysfunction, Insulin resistance

## Abstract

Type 2 diabetes (T2D), a growing global health concern, is often exacerbated by obesity and is linked to severe complications. Serpins, a superfamily of serine protease inhibitors, that includes SerpinA3 in humans and its homolog SerpinA3k in mice. SerpinA3k regulates angiogenesis, reactive oxygen species (ROS) production, inflammation, and fibrosis particularly in the context of experimental ocular injury. Furthermore, we have previously demonstrated that urinary SerpinA3 serves as an early biomarker of kidney damage, including diabetic nephropathy; however, its functional role in renal damage, obesity, and T2D remains largely unexplored. This study explored the impact of SerpinA3k deficiency on obesity and T2D and its associated metabolic dysfunctions. Wild-type (WT, *n* = 27) and SerpinA3k knockout (KO *n* = 26) male mice were randomly assigned to three groups: standard chow diet (SD), high-fat diet (HFD) to induce obesity, and HFD combined with streptozotocin to induce T2D (D2). All the mice were followed and studied after 27 weeks. As expected, WT + D2 mice presented severe hyperglycemia, hyperinsulinemia, increased fat tissue, visceral adipocyte hypertrophy, and renal hyperfiltration. In sharp contrast, diabetic SerpinA3k-deficient mice were protected against these alterations, displaying improved glycemic control, greater pancreatic insulin content, reduced insulin resistance, and favorable adipocyte size remodeling characterized by a lower proportion of medium and large adipocytes. Additionally, these mice showed preserved lipolytic function, and attenuated renal hyperfiltration. Our findings indicate that targeting SerpinA3k mitigates hyperglycemia and insulin resistance, preserves adipose tissue functionality, and potentially prevents metabolic complications. These results highlight SerpinA3k as a promising therapeutic target for T2D.

**Supplementary Information:**

The online version contains supplementary material available at 10.1007/s00018-025-05922-3.

## Introduction

The global prevalence of diabetes is currently estimated at 10.5%, with projections indicating an increase to 12.2% by 2045. As a major risk factor for mortality, diabetes contributed to approximately 6.7 million adult deaths in 2021 due to related complications. Among all diabetes cases, 90% are classified as type 2 diabetes (T2D) [1]. Over 80% of individuals with T2D are either obese or overweight, highlighting obesity as a major contributor to T2D development [2].

Diabetes is associated with numerous microvascular complications, including diabetic retinopathy and diabetic kidney disease (DKD). These complications arise from metabolic dysregulation, such as dyslipidemia and insulin resistance, driven by chronic inflammation and hyperglycemia, ultimately leading to excessive oxidative stress. As a multifactorial disease, diabetes affects multiple organs including adipose tissue, impairs liver function and induces pathological changes in the retina and kidneys [3–6]. In this context, identifying novel therapeutic targets is essential. Serpins are a superfamily of serine protease inhibitors (SERPINs) [7]. In humans, this family includes 34 members, including SerpinA3 also known as alpha-1-antichymotrypsin (AACT). SerpinA3 is an acute-phase protein primarily synthesized in the liver. Its principal physiological target is neutrophil cathepsin G, although it also inhibits other serine proteases, such as, chymotrypsin, mast cell chymase, and kallikrein. In contrast, the Mouse Genome Sequencing Consortium identified 14 SerpinA3-related genes clustered on mouse chromosome 12E with a high degree of sequence homology; this conservation suggests that these genes originated from a common ancestral gene that is homologous to human SerpinA3 [7, 8]. Among these paralogs, SerpinA3k is often regarded as one of the functionally closest counterparts to human serpinA3 [9–16]. In addition to its inhibitory functions, SerpinA3k regulates numerous pathological mechanisms such as angiogenesis, reactive oxygen species generation, inflammation and fibrosis. These processes contribute to ocular damage, diabetic retinopathy for instance, a common complication in T2D [9–15].

Specifically, Zhang B. et al. demonstrated that SerpinA3k attenuates the overexpression of profibrotic proteins such as fibronectin and connective tissue growth factor (CTGF) in rats with diabetic retinopathy [13]. In addition, we recently showed that SerpinA3 is secreted in HEK-293 cells in response to harmful stimuli, such as nutrient deprivation and/or exposure to hydrogen peroxide [17]. These findings further highlight the involvement of SerpinA3 in conditions of cellular stress. However, most studies focusing on SerpinA3, to date have been conducted in models of retinal and corneal damage, leaving the specific role of SerpinA3k in the context of diabetes and obesity largely unexplored.

In this context, we demonstrated that SerpinA3 serves as an early urinary biomarker for the transition from acute kidney injury (AKI) to chronic kidney disease (CKD) in rats. Furthermore, aberrant urinary SerpinA3 excretion has been observed in patients with diverse kidney pathologies, including lupus nephritis, anti-neutrophil cytoplasmic autoantibody (ANCA)-associated-vasculitis, glomerulosclerosis, and diabetic nephropathy, the latter being a common complication of T2D. In healthy human kidneys, SepinA3 is localized to the cytoplasm of proximal tubular epithelial cells [18, 19]. Under inflammatory conditions such as lupus nephritis and DKD, SerpinA3 translocates to the apical membrane of tubular cells, suggesting a secretion mechanism linked to cellular injury [19, 20]. Notably, urinary SerpinA3 is virtually undetectable in systemic inflammatory diseases without renal involvement, supporting its specificity as a marker for kidney damage [19, 20]. In support of this notion, Fan, Z et al. reported increased SerpinA3 expression in renal tubules from patients with DKD and proposed a potential role in modulating inflammation by inhibiting mast cell proliferation and activation [18]. Similarly, elevated levels of SerpinE1 have been observed in obese individuals with nonalcoholic fatty liver disease [21], a change that is reversed with weight loss [22]. Our recent findings further highlight the relevance of SerpinA3k in a murine model of AKI. SerpinA3k deficiency is associated with increased mRNA levels of antioxidant enzymes, reduced activation of apoptosis pathways and attenuation of the glomerular filtration rate (GFR) decline [23]. Collectively, these observations underscore the importance of tightly regulating SerpinA3 expression during pathophysiological stress. However, the role of SerpinA3k in the context of diabetes and its complications remain poorly defined. In the present study, we explored the contribution of SerpinA3k to diabetes progression and its impact on target organs injury, including diabetic kidney disease, via a murine model of SerpinA3k knockout.

## Materials & Methods

All the animal experiments were conducted in strict accordance with the NIH Guide for the Care and Use of Laboratory Animals and the Mexican Federal Regulation for Animal Reproduction, Care, and Experimentation (NOM-062-ZOO-2001). This study was approved by the Animal Care and Use Committee of the Instituto Nacional de Ciencias Médicas y Nutrición Salvador Zubirán (NMM-1984-19-22-1). As we previously described and characterized, SerpinA3k(-/-) knockout (KO) mice were obtained from The Jackson Laboratory (https://www.jax.org/strain/031522) [23]. The mice were housed 2–3 per cage in ventilated racks (NexGen™ Mouse 500 Ecoflo, Allentown), on a 12:12 h light/dark cycle at a temperature of 20–21 °C and a humidity of 29–33%. All the mice were given *ad libitum* access to water and their corresponding diets.

### Mouse Diabetes Model

**Fig. 1:**
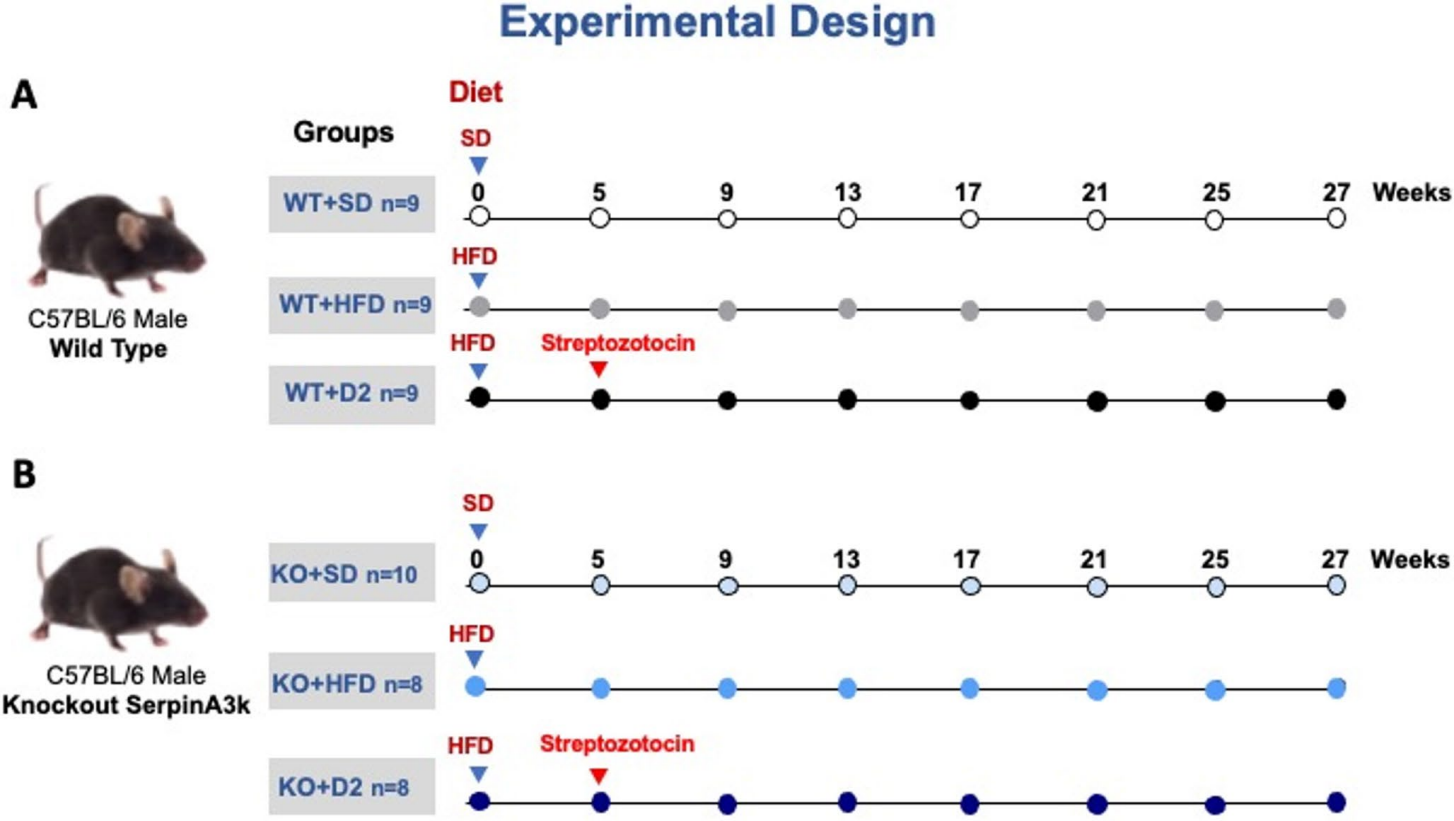
Schematic representation of the experimental protocol used in this study. A total of 27 male wild-type (WT) and 26 male SerpinA3k knockout (-/-) mice were included. After weaning, mice of each genotype were randomly assigned to one of three groups: standard diet (SD), high-fat diet (HFD), or HFD plus streptozotocin (STZ) to induce diabetes (D2). Mice in the WT+D2 and KO+D2 groups were fed a HFD for 5 weeks prior to receiving STZ injections. (**A**) Wild type groups: WT+SD (n=9), WT+HFD (n=9), and WT+D2 (n=9), and (**B**) SerpinA3k groups: KO+SD (n=10), KO+HFD (n=8), and KO+D2 (n=8)

SerpinaA3K(−/−) knockout mice with a C57BL/6NJ genetic background (for more details: https://www.jax.org/strain/031522) were obtained from The Jackson Laboratory. They were intercrossed, and the studied animals were in the third or fourth generation. A total of 53 postweaning male mice were used: 27 wild-type (WT, [SerpinA3k(+/+)]) and 26 knockout (KO, [SerpinA3k(-/-)]) mice. Mice of each genotype were randomly assigned to three groups: standard chow diet (SD; *n* = 9 WT and 10 KO), high-fat diet (HFD; *n* = 9 WT and 8 KO), and diabetes (D2; *n* = 9 WT and 8 KO) groups, as is shown in Fig. 1. Randomization was performed via the GraphPad random number generator, which is available online. The composition and characteristics of each diet have been described previously [24] (for further details, see Table S1). For diabetes induction, the mice were first fed a HFD for 5 weeks. Thereafter, the mice received 100 mg/kg of streptozotocin (STZ) dissolved in citrate buffer (0.1 M pH 4.5) and were fed a HFD throughout the study (Fig. 1), as previously described [25]. Prior to STZ injection, the mice were fasted for 6 h. Fourteen days after STZ administration, body weight was recorded at baseline and then monitored every four weeks thereafter. The mice animals were subsequently monitored for an additional 22 weeks. Glucose levels were measured monthly via a glucometer, with blood drawn from the tip of the tail after a 6 h fast. Six out of nine WT + D2 mice and three out of twelve KO + D2 mice with glucose levels exceeding 450 mg/dL received insulin (2U glargine, Germany) to prevent ketosis. Each month, the mice were placed in metabolic cages for 18 h for urine collection. Body composition was evaluated through magnetic resonance imaging (MRI) (EchoMRI; Echo Medical Systems, Houston, TX, USA). After 27 seven weeks or 22 weeks after STZ administration, the mice were fasted for 6 h before euthanasia during which the GFR was assessed. Briefly, the mice were sedated with intraperitoneal sodium pentobarbital (50 mg/kg) and blood samples were collected via cardiac puncture. The blood was centrifuged (1500 g) for 20 min and the serum was stored at −80 °C. Finally, the retroperitoneal white adipose tissue (rWAT), liver, pancreas, and kidneys were isolated. The tissues were stored at −70 °C for molecular and biochemical analysis, and small pieces of kidney, liver and rWAT tissues were fixed in 4% formaldehyde and then stored in holding buffer until paraffin inclusion.

### Serum biochemistry parameters

At the end of the study, the serum glucose (Cat. No. GPSL-0507), triglycerides (Cat. No. TGML-0507), and cholesterol (Cat. No. CHSL-0507) were measured (ELITech Clinical Systems, Sees, France), as well as glycerol (Sigma, St. Louis, MO, USA). Insulin (Mouse, Cat. No. 80-INSMSU-E01,), adiponectin (Mouse, Cat. No. 47-ADPMD-E01), leptin (Mouse, Cat. No. 22-LEPMS-E01), and interleukin-6 (Cat No. RABO308-1) were measured via ELISA (ALPCO, Salem, NH, USA). Systemic insulin resistance was calculated based on glucose and insulin concentrations through the HOMA-IR index ((glucose mmol/l x insulin µU/l)/22.5) [26], and the lipolysis index was calculated according to the following formula (glycerol µM/(adipose tissue g/body weight g)) [27].

### Pancreatic insulin immunohistochemistry

Immunohistochemistry staining was conducted via the ultraView Universal DAB detection kit (Ventana-Roche) following the manufacturer’s instructions. Briefly, 4 μm thick paraffin-embedded sections of pancreas samples were prepared. They were fixed on kling-On charged glass slides (Biocare Medical) and subjected to deparaffinized through xylene and decreasing series of ethanol as per standard procedures. Antigen retrieval was heat-induced by immersing slides in Diva Decloaker (Biocare Medical) and boiled for 20 min in a pressure cooker. Slides were incubated with primary anti-insulin antibody for 45 min (1:500, Cell Signaling, Cat. No. #3014) and then incubated with the HRP Multimer for 15 min. The reaction was stopped after 1 min and counterstaining was performed with hematoxylin. At least five islets per slide were identified by morphology and digitalized in a blinded fashion using a camera incorporated to a Nikon light microscope. Immunohistochemistry positivity was quantified using NIS-Elements software (Mellville NY, USA, and the percentage of insulin-stained islet area was recorded.

### Adipose, Renal, and hepatic tissue proteins expression assessment

Liver and renal tissues were homogenized in HEPES lysis buffer (50 mM HEPES pH 7.4, 250 mM NaCl, 5 mM EDTA, 0.1% NP-40) with protease inhibitors (Merck, Cat. No. 11697498001), while rWAT samples were processed in RIPA buffer (25 mM Tris-HCl, 150 mM NaCl, 1% Igepal, 0.1% SDS 0.1% and 1% sodium deoxycholate) supplemented with protease inhibitor cocktail (Complete Mini, Roche, Germany Cat. No,11836153001) and phosphatase inhibitors. Proteins were denatured, separated by SDS-PAGE (8.5% or 12% acrylamide), and transferred to PVDF membranes. Blots were blocked with 5% non-fat milk and incubated overnight at 4 °C with primary antibodies against SerpinA3k (1:1,000; Cat. 55480-1-AP, Proteintech, IL. USA), pAKT2 Ser474 (1,2,000; Cat. No, 8599 S, Cell Signaling, MA, USA), totalAKT2 (1:2,000; Cat. No. 3063 S, Cell Signaling, MA, USA) and interleukin 6 (1:1000, Cat. No. SABS700632, Sigma, USA). For sequential detection, membranes were stripped using Re-Blot Plus (Millipore, Cat. No, 2504). Detection was performed using HRP-conjugated goat anti-rabbit IgG (1:15,000, Jackson ImmunoResearch.), and an iBright FL1500 system (Thermo Fisher). Protein normalization was done using β-actin for liver and kidney, and total protein staining (Coomassie blue) for rWAT. Urinary SerpinA3k was assessed with 10 µL of urine via Western blot, as described as above.

### mRNA expression analysis by Semi-quantitative RT-PCR

Total kidney and liver RNA was extracted with TRIzol reagent (Ambiol) and RNA integrity was verified through agarose gel electrophoresis. Complementary DNA (cDNA, Invitrogen. Cat. No. 28025-013) was synthetized via reverse transcription. Gene expression was evaluated through specific probes (Table S2) in a QuantStudio 5 real-time PCR system (Life Technologies). Whereas liver mRNA levels were quantified using SYBR green real-time PCR system (CFX96, Bio-Rad, Table S3) and the relative gene expression was calculated according to the equation for relative expression [28] and normalized with two housekeeping genes, m36B4 and b-2 microglobulin.

### Glomerular filtration rate (GFR) measurement

The GFR was assessed using previously established methods [24, 29]. The mice were anesthetized with sodium pentobarbital (30 mg/kg, i.p.), and a fluorescent transdermal sensor (1727, MediBeacon GmbH, Mannheim, GE) was placed and fixed on the depilated skin. A baseline reading was recorded for 2 min, followed by the administration of 2.1 mg of FITC-sinistrin in 0.06 mL of 0.9% NaCl (FTC-FS001, MediBeacon GmbH, Mannheim, GE) via retroorbital sinus. The clearance rate of FITC-sinestrin was then monitored for at least 1 h to calculate the GFR.

### Histological analysis and size quantification of adipocytes and hepatic lipid droplets

Fixed mouse liver and rWAT samples were embedded in paraffin and sectioned at 4 and 6 μm, respectively, for histological analysis. Sections were stained with hematoxylin and eosin and examined at 200x magnification using an Olympus BX51 microscope. The area of adipocytes and of liver lipid droplets were measured using ImageJ software. The relative frequency distributions areas were calculated, respectively and expressed as percentage of total cells of lipid drops for each experimental group.

### Triglyceride content in the liver

The hepatic lipids were extracted from frozen liver samples using the Folch method [30]. The triglyceride levels were quantified using a colorimetric-enzymatic assay kit (ELITech Clinical Systems, Sees, France) and normalized to the weight of each liver sample analyzed.

### Statistical analysis

All statistical analyses and graphical representations were performed using GraphPad Prism version 9.5.0. For datasets meeting the assumptions for ANOVA, one-way ANOVA was employed, followed by Bonferroni´s multiple comparison test for *post-hoc* analysis. For datasets with nonparametric distribution, the Kruskal-Wallis test was applied, followed by Dunn´s multiple comparison test and were log transformed. Statistical significance was defined as a *p-value* < 0.05.

## Results

### Metabolic evaluation in WT and KO obese and diabetic mice

**Fig. 2:**
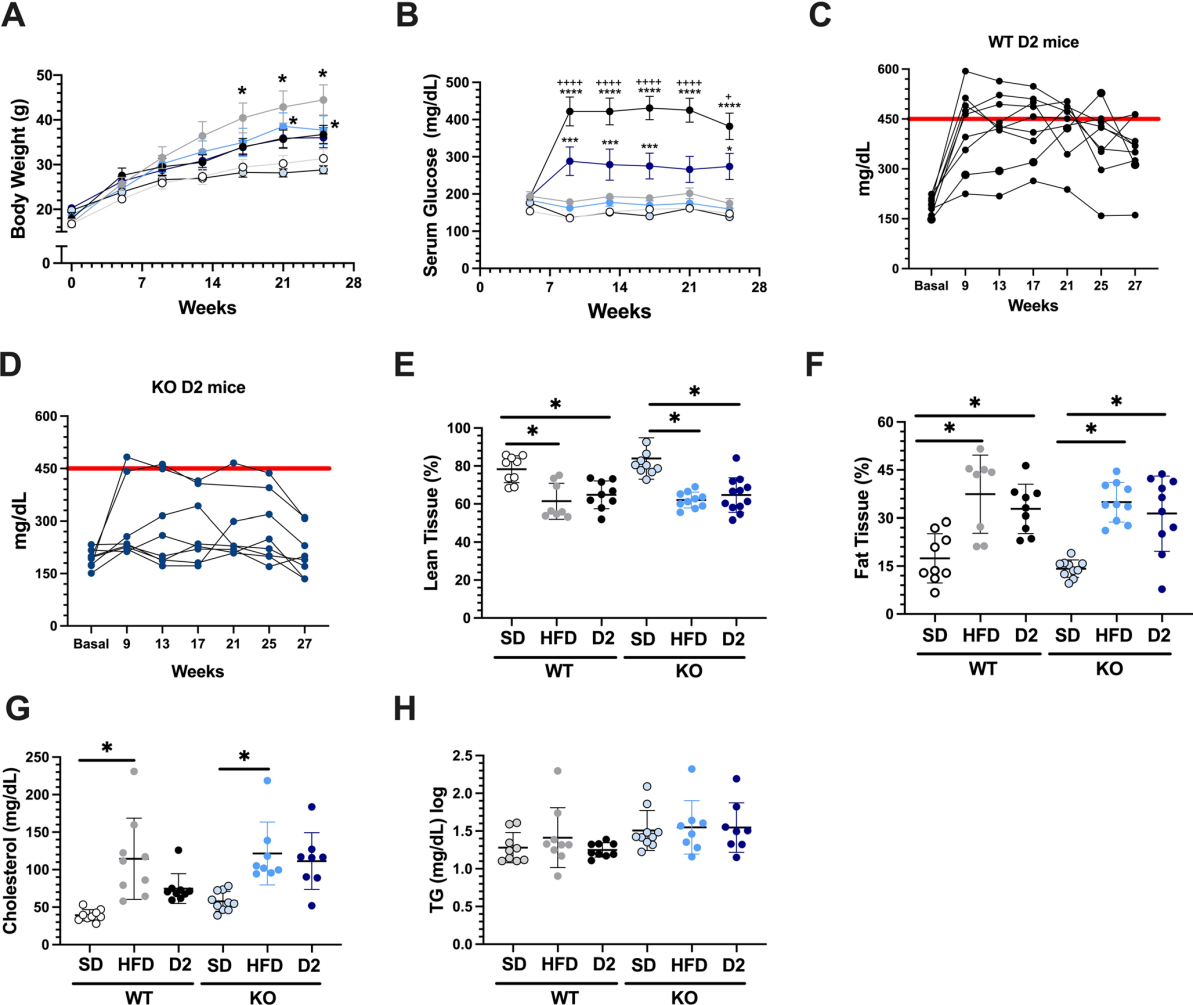
Metabolic evaluation in WT and KO obese and diabetic mice. (**A**) Body weight monitored throughout the follow-up period for: wild-type (WT) or serpinA3k knockout (KO) mice fed a standard chow diet (SD), high-fat diet (HFD), and HFD + streptozotocin (D2), respectively. (**B**) Six-hour fasting blood glucose concentrations measured monthly at 5-, 9-, 13-, 17-, 21-, and 27-weeks*.* (**C**) Glycemic trajectories of individual WT+D2 mice. (**D**) Glycemic trajectories of individual KO+D2 mice. (**E**) Percentage of lean tissue. (**F**) Percentage of fat tissue using magnetic resonance imaging. (**G**) Serum cholesterol and (**H**) serum triglyceride (TG) concentrations at the end of the experiment after eight-hour fasting. Data are presented as mean ± SD. *p<0.05, ***p<0.001, and ****p<0.0001 vs. their respective WT+SD or KO+SD controls as stated; +*p*<0.05, and ++++*p*<0.0001 vs. KO+D2 mice at 9-, 13-, 17-, and 27-weeks, respectively, as stated

Body weight (BW) and glucose concentration throughout the study, together with body composition and cholesterol and triglycerides (TG) levels at the end of the experimental period, are illustrated in Fig. 2. BW gradually increased in the all mice over the 27-weeks period of the study due to their normal growth. However, the WT + HFD group presented greater weight gain, with a statistically significant increase observed since 17-weeks compared to the WT + SD diet. Similarly, the KO + HFD had a significant increase in BW compared with the KO + SD group beginning at week 21 (Fig. 2A). As expected, a single STZ injection administered five weeks after starting the HFD led to a significant and sustained elevation of serum glucose in the WT + D2 group compared with the WT + SD group. The KO + D2 group also developed hyperglycemia; however, their blood glucose levels were significantly lower than those of the WT + D2 group (Fig. 2B). Figures 2C and 2D show the individual glycemic trajectories of WT + D2 and KO + D2 mice, respectively. During the follow-up period, six of nine WT + D2 mice and two of eight KO + D2 mice presented serum glucose levels exceeding 450 mg/dL. These animals received insulin treatment to prevent ketosis and severe hyperglycemia, as indicated by the red line in the graphs. Notably, the WT + D2 mice maintained marked hyperglycemia throughout the study despite insulin administration, a pattern not observed in the SerpinA3k KO mice. These findings indicate that, despite insulin treatment, the metabolic profiles differ significantly between diabetic WT and KO mice, suggesting that SerpinA3k deficiency contributes to the observed metabolic protection. Analysis of body composition revealed that all HFD-fed groups had a lower percentage of lean tissue, and a greater percentage of fat tissue than did the control groups. This effect was not influenced by streptozotocin administration and did not differ between genotypes (Fig. 2E and 2F). Compared with control mice, HFD-fed mice presented increased cholesterol levels across genotypes. While both diabetic groups presented hypercholesterolemia, the difference was not statistically significant compared with that of their respective controls (Fig. 2G). No significant differences in triglyceride levels were observed among the studied groups (Fig. 2H).

### Hyperglycemia and hyperinsulinemia are improved in SerpinA3k knockout mice

**Fig. 3:**
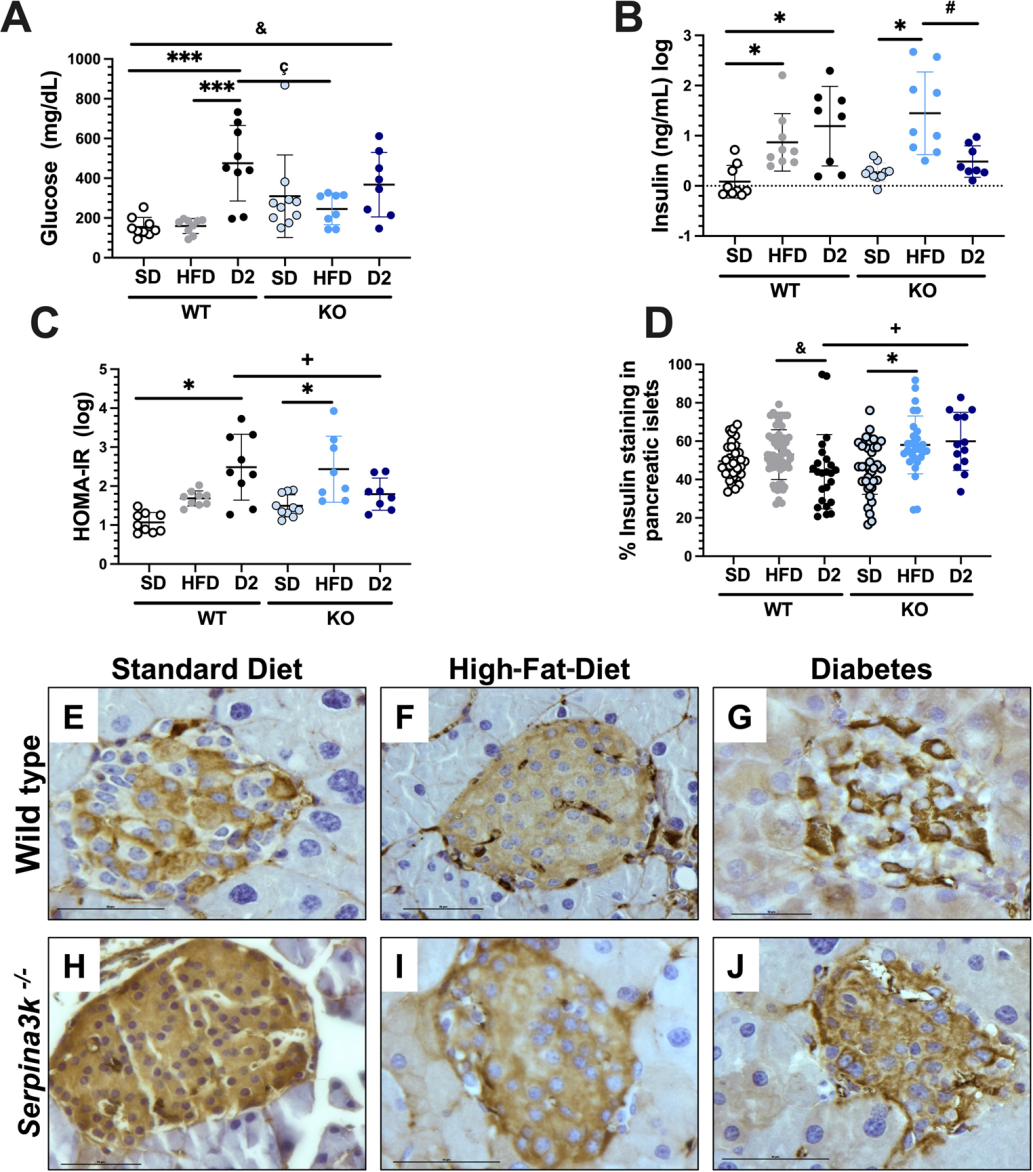
Glucose, insulin, and HOMA-IR levels. Eight-hour fasting glucose at the end of the experiment. (**A**) serum glucose and (**B**) serum insulin concentration was evaluated. (**C**) HOMA-IR assessed at the end of the follow-up period. (**D**) Positive insulin immunostaining in pancreatic islets, whit representative images from (**E**) wild-type mouse on a standard chow diet, (**F**) wild-type mouse on a high-fat diet (HFD), (**G**) wild-type diabetic mouse, (**H**) SerpinA3k knockout mouse on a standard chow diet. (**I**) SerpinA3k knockout mouse on a high-fat diet, and (**J**) SerpinA3k knockout diabetic mouse. Data are presented as mean ± SD, *p<0.05 vs. their respective WT+SD or KO+SD controls**. **#*p*<0.05 KO+HFD vs. KO+D2, +*p*<0.05 WT+D2 vs. KO+D2; &*p*<0.05 WT+HFD vs. KO+D2; ç *p*<0.05 WT+D2 vs. KO+HFD, as stated

At the end of the study, central glucose levels were quantitatively measured, and were similar to the glucose follow-up results obtained with tail-tip glucometer, where the WT + D2 group presented significantly greater values than the WT + SD and WT + HFD groups did. Interestingly, the KO + D2 animals presented lower glucose levels, which were not significant different from those of the KO + SD and KO + HFD groups, but remained significantly greater than the WT + SD and WT + HFD groups (Fig. 3A). In the serum insulin assessment, we found that both the HFD-fed and the WT + D2 groups exhibited hyperinsulinemia, an effect that was bot observed in the KO + D2 group (Fig. 3B). Insulin resistance, as evidenced by the HOMA-IR index, was significantly different between the WT + D2 group and the WT + SD group. However, insulin resistance was not observed in the KO + D2 group compared with the KO + HFD and the WT + D2 groups (Fig. 3C). Furthermore, we observed that in the WT + D2 group there was a reduction in insulin immunostaining in pancreatic islets, whereas in the KO + D2 group, insulin immunostaining was not reduced (Fig. 3E to 3J).

### SerpinA3k deficiency improves adipocyte size and lipolysis

**Fig. 4:**
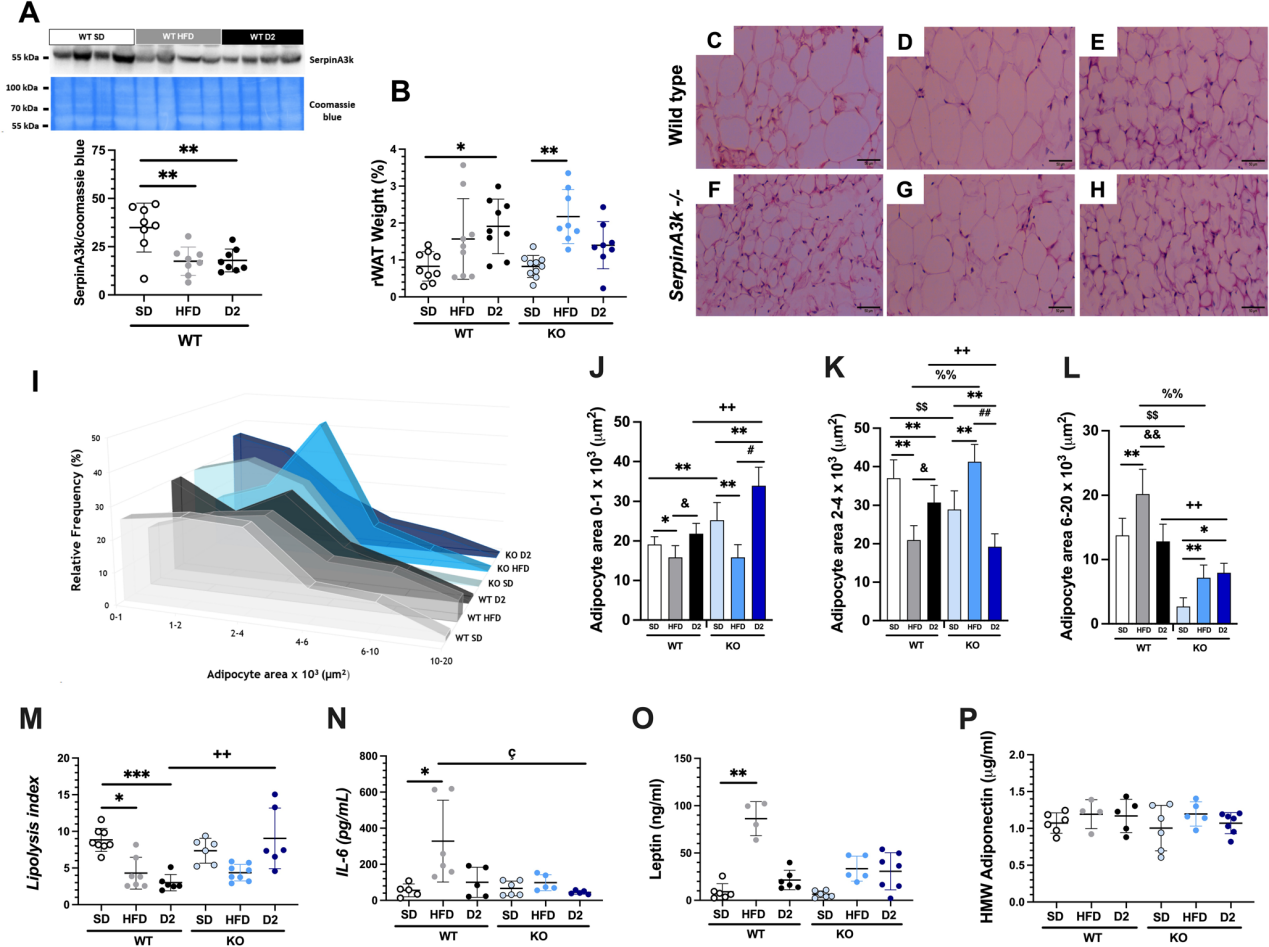
Analysis of SerpinA3k expression and retroperitoneal white adipose tissue (rWAT) parameters. (**A**) Representative blots and densitometric analysis of SerpinA3k abundance in the retroperitoneal rWAT of wild-type (WT) animals fed a standard chow diet (SD), high-fat diet (HFD) or with type 2 diabetes (D2), normalized to Coomassie blue staining. (**B**) Weight of the rWAT tissue in WT and SerpinA3K knockout (KO) mice. (**C **to **H**) Representative histomorphology of rWAT tissue (200X magnification). (**I**) Relative frequency (percentage) of adipocyte areas stratified by size. (**J**) Relative frequency (percentage) of adipocytes ranging from 0 to 1000 m^2^ (**K**) from 2,000 to 4,000 m^2^ (**L**) and from 6,000 to 20,000 m^2^. (**M**) Fasting lipolysis index, (**N**) interleukin-6 (IL-6), (**O**) leptin, and (**P**) high molecular weight (HMW) adiponectin. Data are presented as mean ±SD. Statistical significance: *p<0.05,**p<0.01, and ***p<0.001 vs. their respective WT+SD or KO+SD controls; ++*p*<0.01 WT+D2 vs. KO+D2 mice**; **#*p*<0.05 and ##*p*<0.01 KO+HFD vs. KO+D2; ++*p*<0.01 WT+D2 vs. KO+D2. &*p*<0.05 &&*p*<0.01WT+HFD vs. WT+D2; and %%*p*<0.01, WT+HFD vs. KO+HFD; ç *p*<0.05 WT+D2 vs. KO+HFD, as stated

Interestingly, the expression of SerpinA3k in rWAT was notably reduced in the WT + HFD and WT + D2 groups (Fig. 4A) at the end of the study. SerpinA3k expression was not assessed in the KO groups, as we previously demonstrated the absence of this protein in these mice [23]. Compared with the WT + SD group, the WT + D2 group accumulated significantly more rWAT and there was an increasing trend in the WT + HFD group. However, in the KO mice the increase occurred only in those fed a HFD, whereas the weight of the rWAT from the KO + D2 group remained similar to that of the KO + SD group, indicating that the mice in the KO + D2 group tended to accumulate less rWAT than did the WT + D2 mice (Fig. 4B). Moreover, adipocyte size varied across conditions (Fig. 4C to 4I). Adipocyte size is a key indicator of functionality and insulin sensitivity, with smaller adipocytes being more functional. The frequency of small adipocytes was greater in mice from the KO + SD and KO + D2 groups than in their WT counterparts (Fig. 4J). Conversely, the KO + SD and KO + D2 groups presented a lower frequency of medium-sized adipocytes than the WT + SD and WT + D2 groups did, whereas the KO + HFD group presented an increased frequency compared to the WT + HFD group did (Fig. 4K). Notably, the number of large adipocytes was drastically lower in the SerpinA3k KO mice than in their WT counterparts across all conditions (Fig. 4L). Therefore, the rWAT adipocytes of the KO + D2 group underwent a remodeling process characterized by an increased proportion of small adipocytes, in which the frequency of the small adipocytes (insulin sensitive) increased, and that of medium and large adipocytes decreased. Lipolysis is a key metabolic function of adipose tissue that is negatively regulated by insulin signaling and is frequently impaired in individuals with obesity and T2D. In the WT + HFD and WT + D2 groups, the lipolysis index significantly decreased. Notably, in the KO + D2 mice, the lipolysis index was restored, suggesting a potential regulatory role of SerpinA3k in adipose tissue metabolism (Fig. 4M). Furthermore, these findings underscore a distinct difference in lipolytic activity between the WT + T2D and the KO + T2D groups. As expected, the WT + HFD group presented significant increases in the serum IL-6 (Fig. 4N) and leptin concentrations (Fig. 4O). However, this increase induced by the HFD was abolished in the KO mice (Fig. 4N and O). In contrast, the levels of serum HMW adiponectin were similar among the six experimental groups (Fig. 4P).

### SerpinA3k deficiency improves hyperfiltration in diabetic mice

**Fig. 5:**
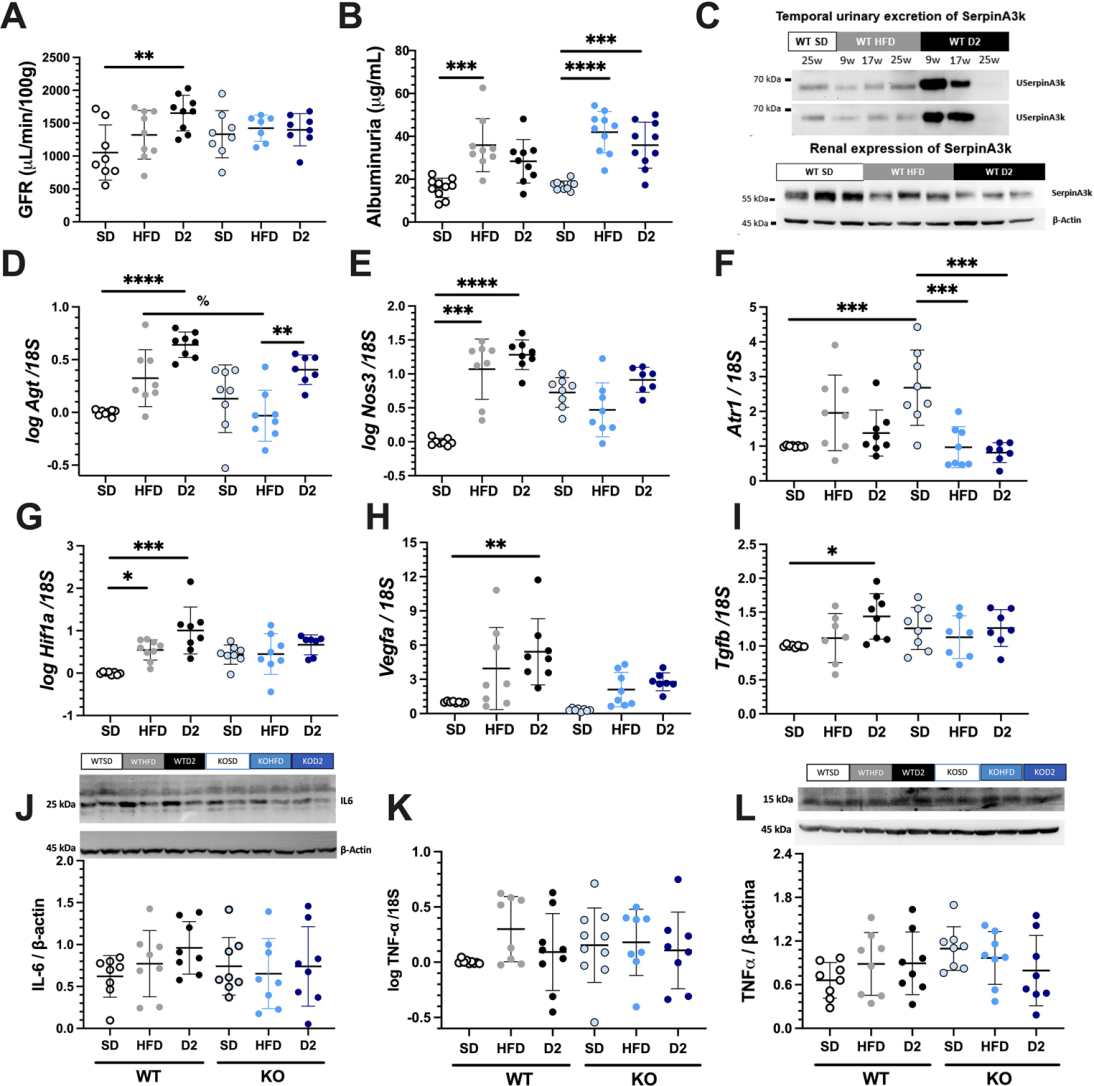
Renal function and intrarenal vasoactive and angiogenic mRNA levels. (**A**) Glomerular filtration rate (GFR) after 27 weeks of follow-up in wild-type (WT) or serpinA3K knockout mice fed standard chow diet (SD), high-fat diet (HFD), or HFD + type 2 diabetes (D2), respectively. (**B**) Albuminuria concentration measured at 27 weeks. (**C**) Representative Western Blots: the upper panel show urinary serpinA3k excretion form two mice per group at 9, 17, and 25 weeks of the study. The lower panel displays intra-renal serpinA3k expression and -β-actin levels from three mice in the WT+SD, WT+HFD, and WT+D2 groups. Densitometric analysis is provided in Supplementary Figure 1. Relative mRNA abundance of genes coding for of (**D**) *Agt*, (**E**) *Nos3*, (**F**) *Atr1*, (**G**) *Hif1a*, (**H**) *Vegfa*, (**I**) *Tgfb*normalized by *18s,*in wild-type (WT) and serpinA3K knockout mice fed SD, HFD, or HFD+D2, respectively. (**J**) IL-6 expression, (**K**) *Tnfa* normalized by *18s*, and (**L**) TNFα expression. Data are presented as mean ± SD. Statistical significance: *p<0.05, **p<0.01, ***p<0.001, and ****p<0.0001 vs*.* their respective WT+SD or KO+SD controls; and %*p*<0.05, WT+HFD vs. KO+HFD

The GFR was estimated via sinistrin-clearance. Diabetes induction in WT mice resulted in hyperfiltration, as demonstrated by the significant increase in the GFR compared with that in the WT + SD mice. Although the KO + SD group presented higher baseline GFR values than the WT + SD group did, the difference was not statistically significant. Moreover, unlike that in WT + D2 mice, the GFR in KO mice remained unchanged following HFD or STZ treatment (Fig. 5A). Both the obese and diabetic groups exhibited increased albuminuria, however no significant differences were observed between the genotypes (Fig. 5B). Urinary SerpinA3k excretion in WT mice significantly increased at 9- and 17-weeks after HFD or diabetes induction (upper panel Fig. 5C and Supplementary Fig. 1). Interestingly, at the end of the study, renal SerpinA3k expression was markedly reduced in the WT + D2 groups and tended to decrease in the WT + HFD group (lower panel Fig. 5C and Suppl. Figure 1). This reduction may partially explain the absence of urinary SerpinA3k excretion at week 25. Since hyperfiltration was not observed in the KO + D2 group, as in the WT + D2 group, we examined the mRNA levels of vasoactive factors, the Hif1a/Vegf pathway, and *Tgfb*. The WT + D2 group presented statistically significant increases in *Agt* and *Nos3* mRNA levels, whereas this alteration was not present in the KO + D2 group (Fig. 5D and 5E). *Atr1* levels were significantly greater in the KO + SD group than in the WT + SD group, but this difference was not observed in the KO + D2 group (Fig. 5F). Compared with those in the WT + SD group, significant increases in *Hif1a* mRNA levels were detected exclusively in the WT + HFD and WT + D2 groups (Fig. 5G). These changes were reflected in its target protein, as *Vegfa* mRNA levels were significantly greater in the WT + D2 group than the WT + SD group, but not in the KO + HFD and KO + D2 groups (Fig. 5H). The *Tgfb* mRNA levels were significantly elevated in the WT + D2 group (Fig. 5I), but this effect was not observed in the KO + D2 group. However, this increase did not correlate with greater tubulointerstitial fibrosis, as histological analysis of Masson’s trichrome-stained kidney sections did not reveal clear evidence of renal fibrosis at this stage of the study (data not shown). Finally, renal inflammation was assessed by evaluating IL-6 and TNFα expression in renal tissue. Although IL6 expression appeared greater in the WT + D2 groups compared to the WT + SD group (0.96 ± 0.11 vs. 0.62 ± 0.09, respectively, *p* = 0.15), this difference did not reach statistical significance. Notably, this pattern was not observed in the KO + D2 group (0.73 ± 0.17 vs. 0.74 ± 0.12, respectively) as is shown in the Fig. 5J. Similarly, *Tnfa* mRNA and TNFα protein levels showed a tendency to increase in the WT + HFD and WT + D2 groups, however these differences did not reach statistical significance (Fig. 5 K and L).

### SerpinA3k deficiency induced lipid synthesis and accumulation in the liver

**Fig. 6:**
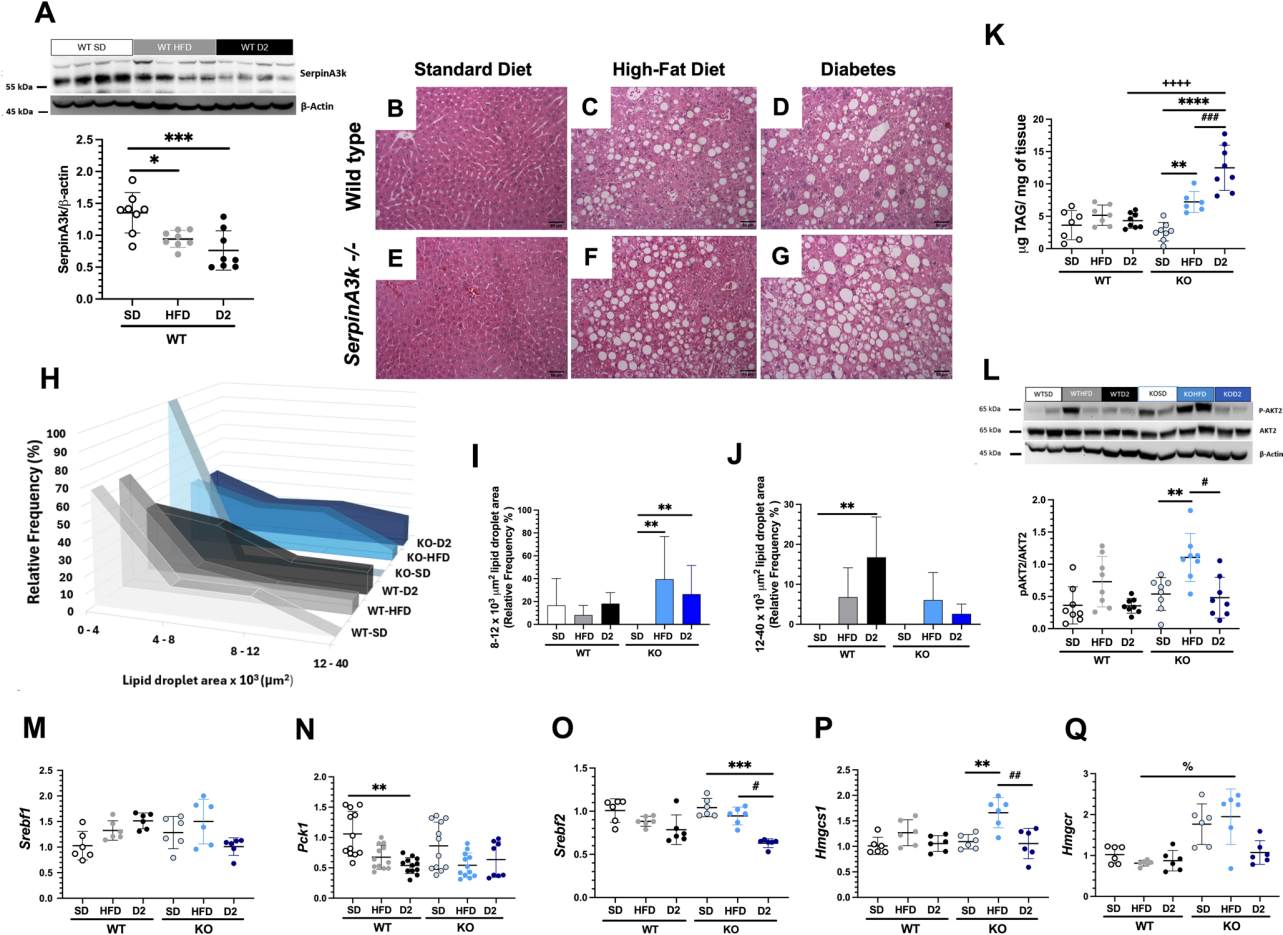
Analysis of hepatic SerpinA3k expression, lipid accumulation, and related parameters. (**A**) Representative blots and densitometric analysis of SerpinA3k abundance in the liver of wild-type (WT) animals fed a standard chow diet (SD), high-fat diet (HFD) or with type 2 diabetes (D2), normalized by β-actin. (**B** to **G**) Representative hepatic histomorphology images showing lipid droplets (200X magnification) in WT and SerpinA3k knockout (KO) mice on SD, HDF or type 2 diabetes as stated. (**H**) Relative frequency (percentage) of lipid droplets area stratified by sizes. (**I**) Relative frequency (percentage) of lipid droplets ranging from 8000 to 12000 μm^2^. (**J**) Relative frequency (percentage) of lipid droplets ranging from 1^2^000 to 40000 μm^2^. (**K**) Triglycerides content in the liver. (**L**) Representative blots and densitometric analysis of the pAkt2 Se474/Akt ratio in the liver of WT and KO mice, normalized to β-actin. Relative mRNA abundance of genes coding for: (**M**) *Srebp-1c*, (**N**) *Pck1,* (**O**) *Srebp2*, (**P**) *Hmgcs1*, and (**Q**) *Hmgcr CoA* normalized by *m36B4* and *b-2 microglubulin.* Data are presented as mean ± SD. *p <0.05, **p <0.01, ***p <0.001, and ****p<0.0001*vs.* their respective WT+SD or KO+SD controls as stated; ++++*p*<0.0001 WT+D2 vs. KO+D2 mice; #*p*<0.05 and ##*p*<0.01 KO+HFD vs*.* KO+D2; and %*p*<0.05, WT+HFD vs. KO+HFD, as stated

Similar to the findings in adipocytes and renal tissue, serpinA3k levels were notably lower in the livers of the WT + HFD and WT + D2 groups, than in those of the WT + SD group (Fig. 6A). This reduction was associated with intracellular fat accumulation (Fig. 6B to 6G). Quantification of fat droplet infiltration revealed that the relative frequency of medium-sized droplets (8 to 12 × 10^3^ µm^2^) was significantly greater in the KO + HFD and KO + D2 groups, than in the KO + SD control group (Fig. 6H and I). Additionally, the WT + D2 group presented an increase in the frequency of larger lipid droplets (>12 × 10^3^ µm^2^), which was not observed in the KO + D2 group (Fig. 6H and 6J). Interestingly, in the KO + SD group, all lipid droplets were smaller than 8 µm^2^ (Fig. 6H, 6I, and 6J). Accordingly, the KO + HFD and KO + D2 groups presented greater intracellular TG content than the KO + SD group did (Fig. 6K), and the KO + D2 group even presented greater TG hepatic content than did its counterpart, the WT + D2 group. However, only the KO + HFD group presented an elevated proportion of phosphorylated Akt2 under fasting conditions, indicating desensitization of the insulin-mediated lipogenesis pathway (Fig. 6L). In addition to the hepatic lipid accumulation observed in the HFD and D2 groups, the expression of genes involved in *de novo* lipogenesis, such as those coding for the transcription factor SREBP-1c and for the rate-limiting enzyme in glyceroneogenesis and essential for fatty acid esterification phosphoenilpyruvate carboxykinase (PEPCK) [31], remained both unchanged among all the groups (Fig. 6M and 6N) or even decreased in the WT + D2 group (Fig. 6N). Thus, the differences observed in serum glucose (Fig. 2B) do not seem to be attributable to alterations in the expression of PEPCK, which is the first rate-limiting enzyme involved in hepatic gluconeogenesis (Fig. 6N). Finally, hepatic transcription factors (Fig. 6O) and enzymes (Fig. 6P and 6Q) involved in cholesterol synthesis provided minimal insight into the serum cholesterol patterns (Fig. 2E), particularly genes encoding HMG-CoA synthase and HMG CoA reductase (*Hmgcs1* and *Hmgcr*, respectively), which were significantly upregulated in the KO + HFD group (Fig. 6P and 6Q).

## Discussion

This study demonstrates that SerpinA3k deficiency provides significant protection against T2D, and several associated metabolic complications. As expected, WT + D2 mice developed severe hyperglycemia, hyperinsulinemia, increased fat tissue, visceral adipocyte hypertrophy, and renal hyperfiltration. In sharp contrast, SerpinA3k-deficient mice were substantially protected from these alterations, exhibiting improved glycemic control, higher pancreatic insulin content, reduced insulin resistance, decreased adipocyte size with fewer medium and large adipocytes, preserved lipolytic function, and attenuated renal hyperfiltration. Collectively, these findings identify SerpinA3k as a key regulator of metabolic homeostasis and a potential therapeutic target in T2D.

Adipose tissue dysfunction, which is characterized by adipocyte hypertrophy, chronic inflammation, and altered secretion of leptin, plays a critical role in the development of insulin resistance and metabolic complications in individuals with T2D [32–34]. Here, we showed that WT + HFD and WT + D2 mice presented larger adipocytes and elevated serum leptin. Higher levels of proinflammatory cytokine IL-6 were also observed in the WT + HFD group. In contrast, the KO + HFD and KO + D2 mice presented smaller adipocytes, and reduced serum IL6 levels, despite facing similar dietary challenges. Adipocyte hypertrophy leads to hypoxia, which triggers the release of proinflammatory adipokines and the recruitment of immune cells [35]. This inflammatory environment disrupts insulin signaling pathways, worsening insulin resistance [34, 36]. By maintaining smaller adipocytes and reducing inflammation, SerpinA3k KO mice may help preserve insulin sensitivity and metabolic homeostasis in mice. In contrast, secreted HMW adiponectin remained unchanged across the experimental conditions. The stability of circulating adiponectin levels in obese mice may be attributed to fat distribution and adipocyte characteristics. Adiponectin is predominantly secreted by subcutaneous fat, whereas visceral fat, which is more strongly associated with metabolic dysfunction, contributes less to this process [37]. If obesity in these mice results primarily from subcutaneous fat expansion, adiponectin production may remain stable. Moreover, adiponectin levels correlate more closely with the total number of adipocytes rather than with their size [38]. Therefore, the observed weight gain in these mice was likely driven by subcutaneous adipose tissue hyperplasia.

The protective effects observed in SerpinA3k KO mice highlight its role as a key modulator in the balance between glucose metabolism and inflammation. The improvement in adipose tissue functionality may play a role, as chronic inflammation is known to impair insulin action [39]. Compared with those in WT + HFD mice, normal systemic IL-6 levels in KO + HFD and KO + D2 mice, suggest that SerpinA3k deficiency mitigates systemic inflammation; this is critical, as inflammation is a major driver of insulin resistance and diabetic complications [4, 12] Intesryingly, SerpinA3k expression was significantly reduced in the liver, renal, and adipose tissues of WT + HFD and WT + D2 mice (Figs. 4 A and 5C and 6A). We hypothesize that this downregulation may represent an adaptive response aimed at mitigating tissue damage or preserving metabolic homeostasis under pathological conditions. However, further studies are necessary to validate this assumption and to elucidate the underlying regulatory mechanisms.

Insulin is continuously synthesized, and its secretion is tightly regulated through glucose-stimulated exocytosis. In WT + D2 mice, reduced pancreatic insulin immunostaining likely reflects increased secretory demand rather than impaired production, as evidenced by elevated circulating insulin levels. This compensatory hypersecretion is a hallmark of early T2D, driven by insulin resistance, and ultimately contributes to β-cell stress and dysfunction [40]. Interestingly, the KO + D2 mice presented improved insulin sensitivity and better glucose homeostasis. These effects appear to be linked to reduced systemic inflammation. Given the known inhibitory effects of inflammatory cytokines such as IL-6 and TNFα on insulin signaling, these findings suggest that SerpinA3k expression contributes to insulin resistance through proinflammatory mechanisms. Thus, in addition to its previously described noncanonical roles in ocular disease [41, 42], SerpinA3k may exert tissue-specific effects that modulate metabolic inflammation and insulin action, an area that deserves further in-depth study.

Diabetic nephropathy, a leading cause of end-stage renal disease, is characterized by albuminuria, glomerulosclerosis, and initial phase of hyperfiltration followed by a progressive decline in renal function [43]. In this study, WT + D2 mice presented greater values of GFR, increased albuminuria and abnormal urinary SerpinA3k excretion during the early stages of hyperglycemia, which progressively diminished over the follow-up period. By the end of the study, hyperfiltration was linked to the downregulation of SerpinA3k in the kidney. These findings suggest that SerpinA3k plays a significant role in the onset of DKD, as its early upregulation aligns with previous observations in other diabetic models [44, 45]. Moreover, hyperfiltration in the WT + D2 mice may be partially explained by the significant elevation in *Nos3* mRNA levels, suggesting greater nitric oxide generation and subsequent renal vasodilation. Additionally, increased *Agt*, could contribute to glomerular hypertension through efferent arteriolar constriction. Interestingly, these alterations were not observed in the KO + D2 mice. Instead, the KO + D2 mice maintained stable GFR and showed no upregulation of these genes. Although hyperfiltration was not associated by differences in albuminuria between genotypes, is very plausible that a longer follow-up (beyond 9 months) could reveal more pronounced diabetic nephropathy in WT + D2 mice than in KO + D2 mice. However, further studies are needed to validate this hypothesis. Also, the observed differences in renal function between genotypes persisted despite the known nephrotoxic effects of STZ, which are typically associated with high STZ doses (e.g., 150 mg/kg) [46, 47]. Furthermore, increased expression of *Hif1a and Vegfa* in the WT + D2 group could contribute to aberrant angiogenesis in the kidneys [48], while the decreased expression of *Agtr1* in KO + D2 mice suggests reduced angiotensin II-mediated fibrosis progression [49, 50]. Collectively, these findings imply that SerpinA3k deficiency confers renal protection by mitigating pathways involved in DKD progression.

Hepatic steatosis, a common feature of metabolic syndrome and T2D, is characterized by excessive lipid accumulation in hepatocytes [51]. Histological analysis revealed that WT + D2 mice developed marked hepatic steatosis, with increased lipid droplet size. In an apparently paradoxical finding, the livers of the KO + HFD and KO + D2 mice presented greater fat accumulation thar did their WT counterparts: this is particularly evident in the TG content and the increased size of the medium lipid droplets. Interestingly, although SREBP-1 expression was unchanged, a complex mechanism potentially independent of *de novo* lipogenesis through SREBP-1c transcriptional was introduced. Moreover, SREBP-1c-independent lipogenic mechanisms may occur via a reduction in PEPCK abundance [52]. The reduction in the expression of *pck1* may shift hepatic metabolism toward a more lipogenic state, favoring fat storage in the liver. Liver-specific PEPCK knockout mice develop hepatic steatosis after fasting, characterized by a marked increase in hepatic TG content and lipid droplet accumulation, despite the upregulation of various genes encoding enzymes involved in free fatty acid oxidation [52]. In the WT + HFD and WT + D2 mice, hepatic SerpinA3k abundance progressively decreases, accompanied by an increase in intrahepatic TG and a reduction in PEPCK mRNA levels, which may contribute to increased hepatic fat accumulation, suggesting that disruption in PEPCK expression could be a key driver of hepatic lipid accumulation in these metabolic states. Increasing evidence suggests that insulin resistance may be substrate dependent, either glucometabolic or lipometabolic, and tissue-specific [53, 54]. This finding underscores that an improvement in adipose tissue metabolism does not necessarily entail hepatic improvements, and vice versa. However, more studies are needed to determine how SerpinA3k modulates hepatic steatosis. Chronic elevation of free fatty acids (FFAs), primarily derived from dysfunctional adipose tissue, is known to induce peripheral insulin resistance, particularly in skeletal muscle, and to impair pancreatic β-cell function. In KO + D2 mice, the presence of more metabolically functional adipose tissue likely contributes to improved insulin sensitivity and the preservation of pancreatic insulin content, collectively resulting in better glycemic control compared than in WT + D2 mice.

This study also highlights the complex tissue-specific effects of SerpinA3k deficiency, particularly on hepatic lipid metabolism, warranting further investigation. The limitations of this study include the following: (1) the lack of inclusion of female mice due to respiratory dysfunction and animals with advanced renal damage; (2) insulin administration was required in most WT + D2 mice; however, despite this intervention, these mice presented persistently greater glycemia than in KO + D2 mice, indicating clear metabolic differences between the genotypes; (3) scarce investigation into the mechanisms of metabolic regulation of glucose and adipose tissue, and (4) in regard to SerpinA3k role in hepatic steatosis, NF-κB signaling and PPAR-gamma were not investigated.

Taken together, our findings suggest that SerpinA3k deficiency confers metabolic protection in diabetic mice, not through changes in total fat mass or adiponectin levels, but by improving glycemic control and restoring adipocyte metabolic function. This is evidenced by improved adipocyte morphology, a surrogate marker of insulin sensitivity and lipolytic capacity, as well as improved systemic insulin responsiveness. Furthermore, SerpinA3k deficiency appears to mitigate diabetes-associated complications. These results underscore SerpinA3k as a promising therapeutic target for T2D and its associated metabolic disorders.

## Supplementary Information

Below is the link to the electronic supplementary material.

Supplementary Material 1 (PDF 41.8 KB)

Supplementary Material 2 (PDF 156 KB)

Supplementary Material 3 (PDF 4.57 MB)

## Data Availability

All data reported in this paper will be shared by the lead contact upon request.

## References

[1] Ogle GD, James S, Dabelea D, Pihoker C, Svennson J, Maniam J, Klatman EL, Patterson CC (2022) Global estimates of incidence of type 1 diabetes in children and adolescents: results from the International Diabetes Federation Atlas, 10th edition. Diabetes Res Clin Pract 183:109083. 10.1016/j.diabres.2021.109083 34883188 10.1016/j.diabres.2021.109083

[2] Campos-Nonato I, Galvan-Valencia O, Hernandez-Barrera L, Oviedo-Solis C, Barquera S (2023) Prevalencia de obesidad y factores de riesgo asociados en adultos mexicanos: resultados de la Ensanut 2022. Salud Publica Mex 65:s238–s247. 10.21149/14809 38060949 10.21149/14809

[3] Li Y, Liu Y, Liu S, Gao M, Wang W, Chen K, Huang L, Liu Y (2023) Diabetic vascular diseases: molecular mechanisms and therapeutic strategies. Signal Transduct Target Ther 8:152. 10.1038/s41392-023-01400-z 37037849 10.1038/s41392-023-01400-z PMC10086073

[4] Lu Y, Wang W, Liu J, Xie M, Liu Q, Li S (2023) Vascular complications of diabetes: a narrative review. Medicine (Baltimore) 102:e35285. 10.1097/MD.0000000000035285 37800828 10.1097/MD.0000000000035285 PMC10553000

[5] Mohandes S, Doke T, Hu H, Mukhi D, Dhillon P, Susztak K (2023) Molecular pathways that drive diabetic kidney disease. J Clin Invest. 10.1172/JCI165654 36787250 10.1172/JCI165654 PMC9927939

[6] Tuttle KR, Agarwal R, Alpers CE, Bakris GL, Brosius FC, Kolkhof P, Uribarri J (2022) Molecular mechanisms and therapeutic targets for diabetic kidney disease. Kidney Int 102:248–260. 10.1016/j.kint.2022.05.012 35661785 10.1016/j.kint.2022.05.012

[7] Sanchez-Navarro A, Gonzalez-Soria I, Caldino-Bohn R, Bobadilla NA (2021) An integrative view of Serpins in health and disease: the contribution of SerpinA3. Am J Physiol Cell Physiol 320:C106–C118. 10.1152/ajpcell.00366.2020 33112643 10.1152/ajpcell.00366.2020

[8] Heit C, Jackson BC, McAndrews M, Wright MW, Thompson DC, Silverman GA, Nebert DW, Vasiliou V (2013) Update of the human and mouse SERPIN gene superfamily. Hum Genomics 7:22. 10.1186/1479-7364-7-22 24172014 10.1186/1479-7364-7-22 PMC3880077

[9] Liu X, Lin Z, Zhou T, Zong R, He H, Liu Z, Ma JX, Liu Z, Zhou Y (2011) Anti-angiogenic and anti-inflammatory effects of SERPINA3K on corneal injury. PLoS ONE 6:e16712. 10.1371/journal.pone.0016712 21304961 10.1371/journal.pone.0016712 PMC3031620

[10] Zhang B, Hu Y, Ma JX (2009) Anti-inflammatory and antioxidant effects of SERPINA3K in the retina. Invest Ophthalmol Vis Sci 50:3943–3952. 10.1167/iovs.08-2954 19324842 10.1167/iovs.08-2954

[11] Hu J, Zhang Z, Xie H, Chen L, Zhou Y, Chen W, Liu Z (2013) Serine protease inhibitor A3K protects rabbit corneal endothelium from barrier function disruption induced by TNF-alpha. Invest Ophthalmol Vis Sci 54:5400–5407. 10.1167/iovs.12-10145 23821192 10.1167/iovs.12-10145

[12] Zhang B, Ma JX (2008) SERPINA3K prevents oxidative stress induced necrotic cell death by inhibiting calcium overload. PLoS ONE 3:e4077. 10.1371/journal.pone.0004077 19115003 10.1371/journal.pone.0004077 PMC2605247

[13] Zhang B, Zhou KK, Ma JX (2010) Inhibition of connective tissue growth factor overexpression in diabetic retinopathy by SERPINA3K via blocking the WNT/beta-catenin pathway. Diabetes 59:1809–1816. 10.2337/db09-1056 20299474 10.2337/db09-1056 PMC2889783

[14] Zheng X, Cui H, Yin Y, Zhang Y, Zong R, Bao X, Ma JX, Liu Z, Zhou Y (2017) Serpina3k ameliorates the corneal oxidative injury induced by 4-hydroxynonenal. Invest Ophthalmol Vis Sci 58:2874–2883. 10.1167/iovs.17-21544 28586911 10.1167/iovs.17-21544

[15] Zhou T, Zong R, Zhang Z, Zhu C, Pan F, Xiao X, Liu Z, He H, Ma JX, Liu Z et al (2012) SERPINA3K protects against oxidative stress via modulating ROS generation/degradation and KEAP1-NRF2 pathway in the corneal epithelium. Invest Ophthalmol Vis Sci 53:5033–5043. 10.1167/iovs.12-9729 22736614 10.1167/iovs.12-9729

[16] Wang L, Li D, Yao F, Feng S, Tong C, Rao R, Zhong M, Wang X, Feng W, Hu Z et al (2025) Serpina3k lactylation protects from cardiac ischemia reperfusion injury. Nat Commun 16:1012. 10.1038/s41467-024-55589-w 39856050 10.1038/s41467-024-55589-w PMC11760901

[17] Sanchez-Navarro A, Murillo-de-Ozores AR, Perez-Villalva R, Linares N, Carbajal-Contreras H, Flores ME, Gamba G, Castaneda-Bueno M, Bobadilla NA (2022) Transient response of serpinA3 during cellular stress. FASEB J 36:e22190. 10.1096/fj.202101912R 35147994 10.1096/fj.202101912R

[18] Fan Z, Gao Y, Jiang N, Zhang F, Liu S, Li Q (2022) Immune-related SERPINA3 as a biomarker involved in diabetic nephropathy renal tubular injury. Front Immunol 13:979995. 10.3389/fimmu.2022.979995 36304455 10.3389/fimmu.2022.979995 PMC9592916

[19] Sanchez-Navarro A, Mejia-Vilet JM, Perez-Villalva R, Carrillo-Perez DL, Marquina-Castillo B, Gamba G, Bobadilla NA (2019) SerpinA3 in the early recognition of acute kidney injury to chronic kidney disease (CKD) transition in the rat and its potentiality in the recognition of patients with CKD. Sci Rep 9:10350. 10.1038/s41598-019-46601-1 31316093 10.1038/s41598-019-46601-1 PMC6637202

[20] Martinez-Rojas MA, Sanchez-Navarro A, Mejia-Vilet JM, Perez-Villalva R, Uribe N, Bobadilla NA (2022) Urinary serpin-A3 is an early predictor of clinical response to therapy in patients with proliferative lupus nephritis. Am J Physiol-Renal Physiol 323:F425–F434. 10.1152/ajprenal.00099.2022 35834275 10.1152/ajprenal.00099.2022

[21] Fuchs A, Samovski D, Smith GI, Cifarelli V, Farabi SS, Yoshino J, Pietka T, Chang SW, Ghosh S, Myckatyn TM et al (2021) Associations among adipose tissue Immunology, Inflammation, exosomes and insulin sensitivity in people with obesity and nonalcoholic fatty liver disease. Gastroenterology 161:968–981e912. 10.1053/j.gastro.2021.05.008 34004161 10.1053/j.gastro.2021.05.008 PMC8900214

[22] Samovski D, Smith GI, Palacios H, Pietka T, Fuchs A, Patti GJ, Nawaz A, Kahn CR, Klein S (2025) Effect of marked weight loss on adipose tissue biology in people with obesity and type 2 diabetes. Diabetes Care. 10.2337/dc24-2739 40208704 10.2337/dc24-2739 PMC12281976

[23] Gonzalez-Soria I, Soto-Valadez AD, Martinez-Rojas MA, Ortega-Trejo JA, Perez-Villalva R, Gamba G, Sanchez-Navarro A, Bobadilla NA (2023) Serpina3k deficiency reduces oxidative stress in acute kidney injury. Int J Mol Sci. 10.3390/ijms24097815 37175519 10.3390/ijms24097815 PMC10177890

[24] Sanchez-Navarro A, Martinez-Rojas MA, Caldino-Bohn RI, Perez-Villalva R, Zambrano E, Castro-Rodriguez DC, Bobadilla NA (2021) Early triggers of moderately high-fat diet-induced kidney damage. Physiol Rep 9:e14937. 10.14814/phy2.14937 34291592 10.14814/phy2.14937 PMC8295594

[25] Glastras SJ, Chen H, Teh R, McGrath RT, Chen J, Pollock CA, Wong MG, Saad S (2016) Mouse models of diabetes, obesity and related kidney disease. PLoS ONE 11:e0162131. 10.1371/journal.pone.0162131 27579698 10.1371/journal.pone.0162131 PMC5006968

[26] Matthews DR, Hosker JP, Rudenski AS, Naylor BA, Treacher DF, Turner RC (1985) Homeostasis model assessment: insulin resistance and beta-cell function from fasting plasma glucose and insulin concentrations in man. Diabetologia 28:412–419. 10.1007/BF00280883 3899825 10.1007/BF00280883

[27] Leijonhufvud BM, Hertel K, Lofgren P (2010) Lipolysis index: evaluation of a new tool for metabolic assessment in epidemiological studies on obesity. Horm Metab Res 42:907–911. 10.1055/s-0030-1267205 20972942 10.1055/s-0030-1267205

[28] Pfaffl MW, Tichopad A, Prgomet C, Neuvians TP (2004) Determination of stable housekeeping genes, differentially regulated target genes and sample integrity: Bestkeeper–excel-based tool using pair-wise correlations. Biotechnol Lett 26:509–515. 10.1023/b:bile.0000019559.84305.47 15127793 10.1023/b:bile.0000019559.84305.47

[29] Sanchez-Navarro A, Martinez-Rojas MA, Albarran-Godinez A, Perez-Villalva R, Auwerx J, de la Cruz A, Noriega LG, Rosetti F, Bobadilla NA (2022) Sirtuin 7 deficiency reduces inflammation and tubular damage induced by an episode of acute kidney injury. Int J Mol Sci. 10.3390/ijms23052573 35269715 10.3390/ijms23052573 PMC8910458

[30] Folch J, Lees M, Sloane Stanley GH (1957) A simple method for the isolation and purification of total lipides from animal tissues. J Biol Chem 226:497–509 13428781

[31] Yu S, Meng S, Xiang M, Ma H (2021) Phosphoenolpyruvate carboxykinase in cell metabolism: roles and mechanisms beyond gluconeogenesis. Mol Metab 53:101257. 10.1016/j.molmet.2021.101257 34020084 10.1016/j.molmet.2021.101257 PMC8190478

[32] Gregor MF, Hotamisligil GS (2011) Inflammatory mechanisms in obesity. Annu Rev Immunol 29:415–445. 10.1146/annurev-immunol-031210-101322 21219177 10.1146/annurev-immunol-031210-101322

[33] Guilherme A, Virbasius JV, Puri V, Czech MP (2008) Adipocyte dysfunctions linking obesity to insulin resistance and type 2 diabetes. Nat Rev Mol Cell Biol 9:367–377. 10.1038/nrm2391 18401346 10.1038/nrm2391 PMC2886982

[34] Sun K, Kusminski CM, Scherer PE (2011) Adipose tissue remodeling and obesity. J Clin Invest 121:2094–2101. 10.1172/JCI45887 21633177 10.1172/JCI45887 PMC3104761

[35] Ye J, Gao Z, Yin J, He Q (2007) Hypoxia is a potential risk factor for chronic inflammation and adiponectin reduction in adipose tissue of ob/ob and dietary obese mice. Am J Physiol Endocrinol Metab 293:E1118–1128. 10.1152/ajpendo.00435.2007 17666485 10.1152/ajpendo.00435.2007

[36] Olefsky JM, Glass CK (2010) Macrophages, inflammation, and insulin resistance. Annu Rev Physiol 72:219–246. 10.1146/annurev-physiol-021909-135846 20148674 10.1146/annurev-physiol-021909-135846

[37] Marcelin G, Gautier EL, Clement K (2022) Adipose tissue fibrosis in obesity: etiology and challenges. Annu Rev Physiol 84:135–155. 10.1146/annurev-physiol-060721-092930 34752708 10.1146/annurev-physiol-060721-092930

[38] Bahceci M, Gokalp D, Bahceci S, Tuzcu A, Atmaca S, Arikan S (2007) The correlation between adiposity and adiponectin, tumor necrosis factor alpha, interleukin-6 and high sensitivity C-reactive protein levels. Is adipocyte size associated with inflammation in adults? J Endocrinol Invest 30:210–214. 10.1007/BF03347427 17505154 10.1007/BF03347427

[39] Donath MY, Shoelson SE (2011) Type 2 diabetes as an inflammatory disease. Nat Rev Immunol 11:98–107. 10.1038/nri2925 21233852 10.1038/nri2925

[40] Esser N, Utzschneider KM, Kahn SE (2020) Early beta cell dysfunction vs insulin hypersecretion as the primary event in the pathogenesis of dysglycaemia. Diabetologia 63:2007–2021. 10.1007/s00125-020-05245-x 32894311 10.1007/s00125-020-05245-x

[41] Shoelson SE, Herrero L, Naaz A (2007) Obesity, inflammation, and insulin resistance. Gastroenterology 132:2169–2180. 10.1053/j.gastro.2007.03.059 17498510 10.1053/j.gastro.2007.03.059

[42] Hotamisligil GS (2006) Inflammation and metabolic disorders. Nature 444:860–867. 10.1038/nature05485 17167474 10.1038/nature05485

[43] Reutens AT (2013) Epidemiology of diabetic kidney disease. Med Clin North Am 97:1–18. 10.1016/j.mcna.2012.10.001 23290726 10.1016/j.mcna.2012.10.001

[44] Takahashi E, Okumura A, Unoki-Kubota H, Hirano H, Kasuga M, Kaburagi Y (2013) Differential proteome analysis of serum proteins associated with the development of type 2 diabetes mellitus in the KK-A(y) mouse model using the iTRAQ technique. J Proteom 84:40–51. 10.1016/j.jprot.2013.03.014 10.1016/j.jprot.2013.03.014 23545169

[45] Takahashi E, Unoki-Kubota H, Shimizu Y, Okamura T, Iwata W, Kajio H, Yamamoto-Honda R, Shiga T, Yamashita S, Tobe K et al (2017) Proteomic analysis of serum biomarkers for prediabetes using the Long-Evans Agouti rat, a spontaneous animal model of type 2 diabetes mellitus. J Diabetes Investig 8:661–671. 10.1111/jdi.12638 28150914 10.1111/jdi.12638 PMC5583949

[46] Like AA, Rossini AA (1976) Streptozotocin-induced pancreatic insulitis: new model of diabetes mellitus. Science 193:415–417. 10.1126/science.180605 180605 10.1126/science.180605

[47] Szkudelski T (2001) The mechanism of Alloxan and streptozotocin action in B cells of the rat pancreas. Physiol Res 50:537–546 11829314

[48] Nangaku M (2006) Chronic hypoxia and tubulointerstitial injury: a final common pathway to end-stage renal failure. J Am Soc Nephrol 17:17–25. 10.1681/ASN.2005070757 16291837 10.1681/ASN.2005070757

[49] Mezzano SA, Ruiz-Ortega M, Egido J (2001) Angiotensin II and renal fibrosis. Hypertension 38:635–638. 10.1161/hy09t1.094234 11566946 10.1161/hy09t1.094234

[50] Liu Y (2006) Renal fibrosis: new insights into the pathogenesis and therapeutics. Kidney Int 69:213–217. 10.1038/sj.ki.5000054 16408108 10.1038/sj.ki.5000054

[51] Li Y, Xu S, Mihaylova MM, Zheng B, Hou X, Jiang B, Park O, Luo Z, Lefai E, Shyy JY et al (2011) AMPK phosphorylates and inhibits SREBP activity to attenuate hepatic steatosis and atherosclerosis in diet-induced insulin-resistant mice. Cell Metab 13:376–388. 10.1016/j.cmet.2011.03.009 21459323 10.1016/j.cmet.2011.03.009 PMC3086578

[52] She P, Shiota M, Shelton KD, Chalkley R, Postic C, Magnuson MA (2000) Phosphoenolpyruvate carboxykinase is necessary for the integration of hepatic energy metabolism. Mol Cell Biol 20:6508–6517. 10.1128/MCB.20.17.6508-6517.2000 10938127 10.1128/mcb.20.17.6508-6517.2000 PMC86125

[53] Calderon-DuPont D, Torre-Villalvazo I, Diaz-Villasenor A (2023) Is insulin resistance tissue-dependent and substrate-specific? The role of white adipose tissue and skeletal muscle. Biochimie 204:48–68. 10.1016/j.biochi.2022.08.021 36099940 10.1016/j.biochi.2022.08.021

[54] Bo T, Gao L, Yao Z, Shao S, Wang X, Proud CG, Zhao J (2024) Hepatic selective insulin resistance at the intersection of insulin signaling and metabolic dysfunction-associated steatotic liver disease. Cell Metab 36:947–968. 10.1016/j.cmet.2024.04.006 38718757 10.1016/j.cmet.2024.04.006

