# Supplementary material for: SerpinA3k Deficiency Ameliorates Experimental type 2 Diabetes
Source: Cell Mol Life Sci. 2025 Dec 2;82(1):434. doi: 10.1007/s00018-025-05922-3 (PMC12672987; doi:10.1007/s00018-025-05922-3)
Supplement: Supplementary file 1 — Supplementary Material 1 (PDF 41.8 KB) [file 18_2025_5922_MOESM1_ESM.pdf]

Table S1. Content of each diet used in the study

Component	Standard Diet (SD)		High Fat Diet (HFD)	
	kcal/g	Energy supply (%)	kcal/g	Energy supply (%)
**Protein**	0.88	22.17	0.94	18.78
**Corn Oil**	0.45	11.34	0.45	8.99
**Animal lard**	0	0	1.8	35.96
**Polysaccharides**	1.24	31.23	0.808	16.14
**Simple sugars**	1.24	31.23	0.808	16.14
**Fiber**	0.16	4.03	0.2	4.00
**Total Energy (kcal/g)**	4.0	---	5.0	---
**Energy From Total Fat (%)**	---	11.34	---	44.95

Table S2. Taqman Probes.

Target genes	Probe gen catalog number
Hif1a	Mm00468869_m1
Gpx1	Mm00656767_g1
Sod2	Mm01313000_m1
Tnf	Mm00443258_m1
Il6	Mm00446190_m1
Tfgb1	Mm01178820_m1
Il10	Mm01288386_m1
Ccl2	Rn00580555_m1
Nfe2l2	Rn00582415_m1
Vegfa	Rn01511602_m1

Table S3. Sequence of primers.

	Primers	
**Target genes**	**Forward**	**Reverse**
**SREBP-1C**	ACTTTTCCTTAACGTGGGCCT	TGAGCTGGAGCATGTCTTCG
**PEPCK**	GTTTGATGCCCAAGGCAACTT	TGCCTTCCCAGTAAACACCC
**SREBP2**	CTGTCGGGTGTCATGGGC	ACAAACTGTAGCATCTCGTCGAT
**HMG CoA reductasa**	CATCCGTGTACGAGTGCCTG	CACAGTCCTTGGATCCTCGC
**HMG CoA sintasa**	GGGTCGGTGGCTATAAAGCTG	GTTCCTCCTTCAGCCACCAAAG
