# Supplementary material for: SerpinA3k Deficiency Ameliorates Experimental type 2 Diabetes
Source: Cell Mol Life Sci. 2025 Dec 2;82(1):434. doi: 10.1007/s00018-025-05922-3 (PMC12672987; doi:10.1007/s00018-025-05922-3)

# SerpinA3k in the LIVER

25 µg of liver tissue

Anti-SerpinA3k, 1:1000 (Cat. No. 55480-1-AP, Proteintech, IL. USA.)

Goat anti-Rabbit IgG 1:5000 (Cat. No. GtxRb-003-DHRPX, ImmunoReagents, NC. USA.)

Anti-β- Actin HRP 1:500,000 (Cat. No. ab49900, Abcam, Cambridge, UK.)

**GEL 1**

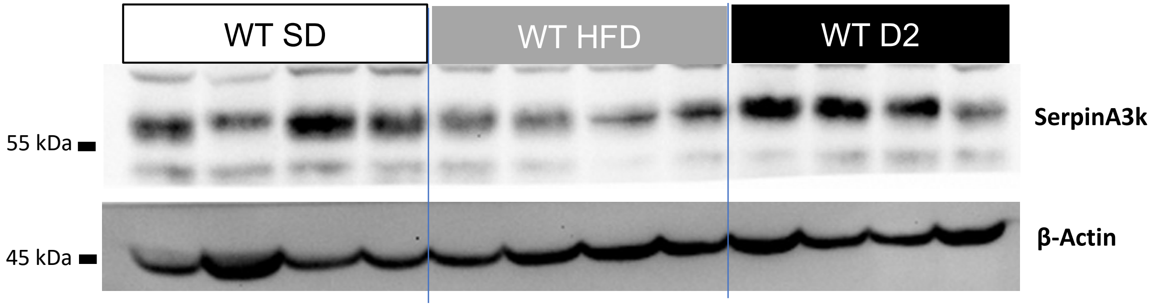


**Duplicate GEL 1**

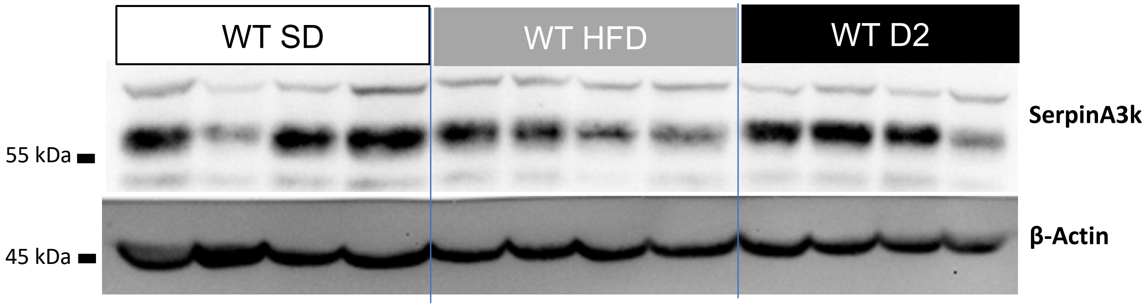


**GEL 2**

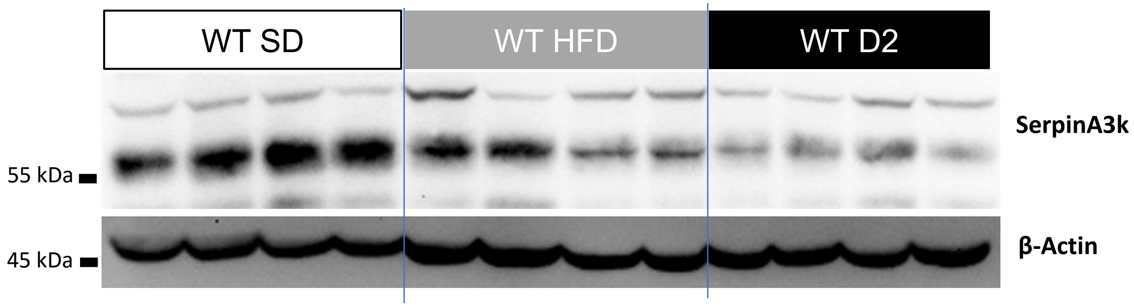


**Duplicate GEL 2**

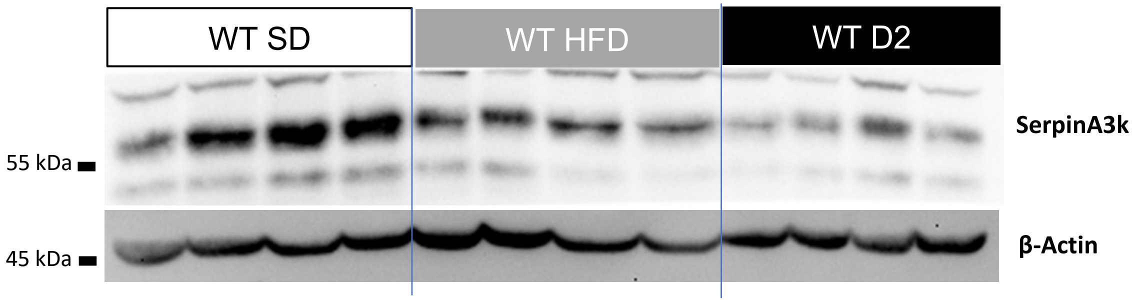

# p-AKT2 and AKT2 in the LIVER

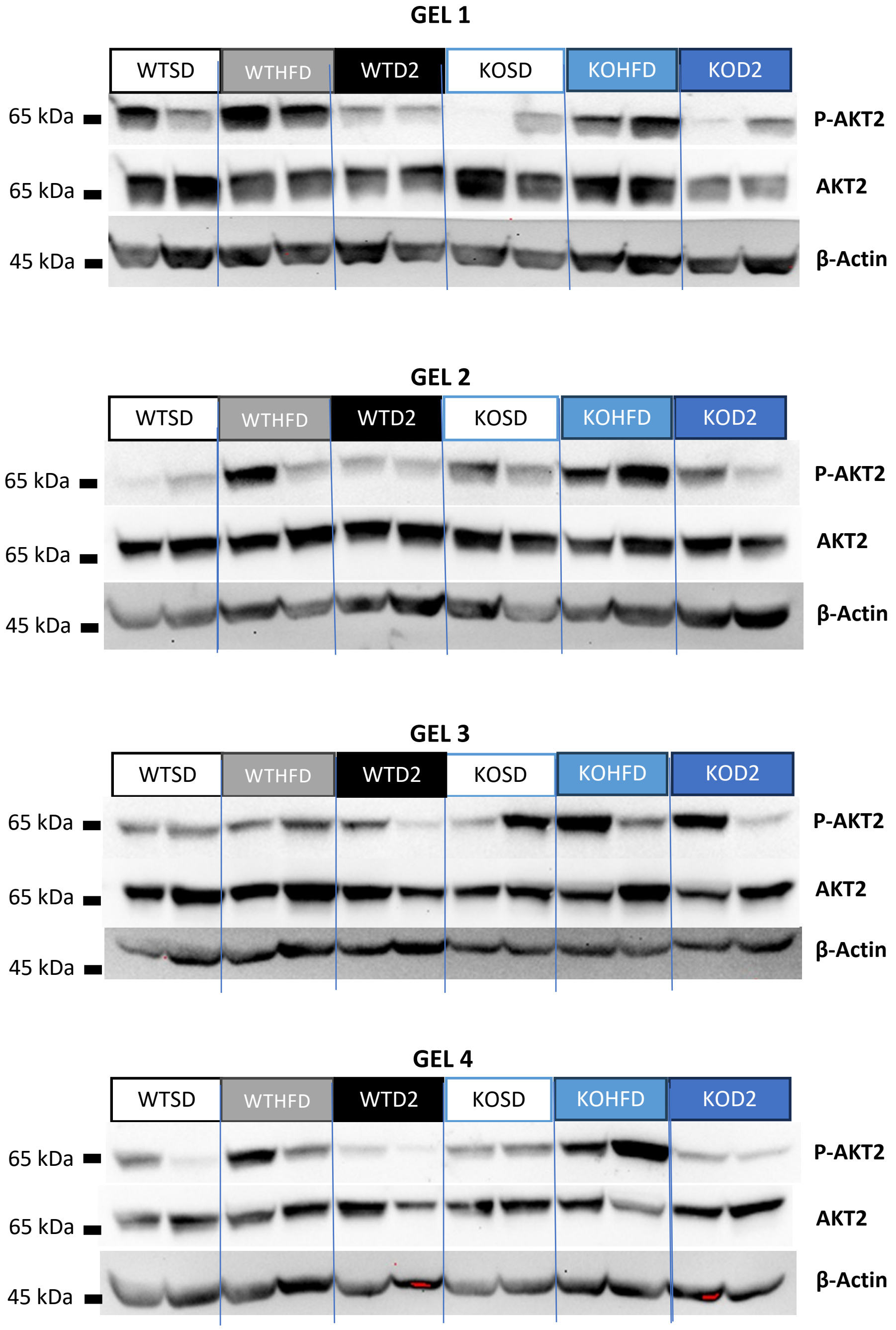


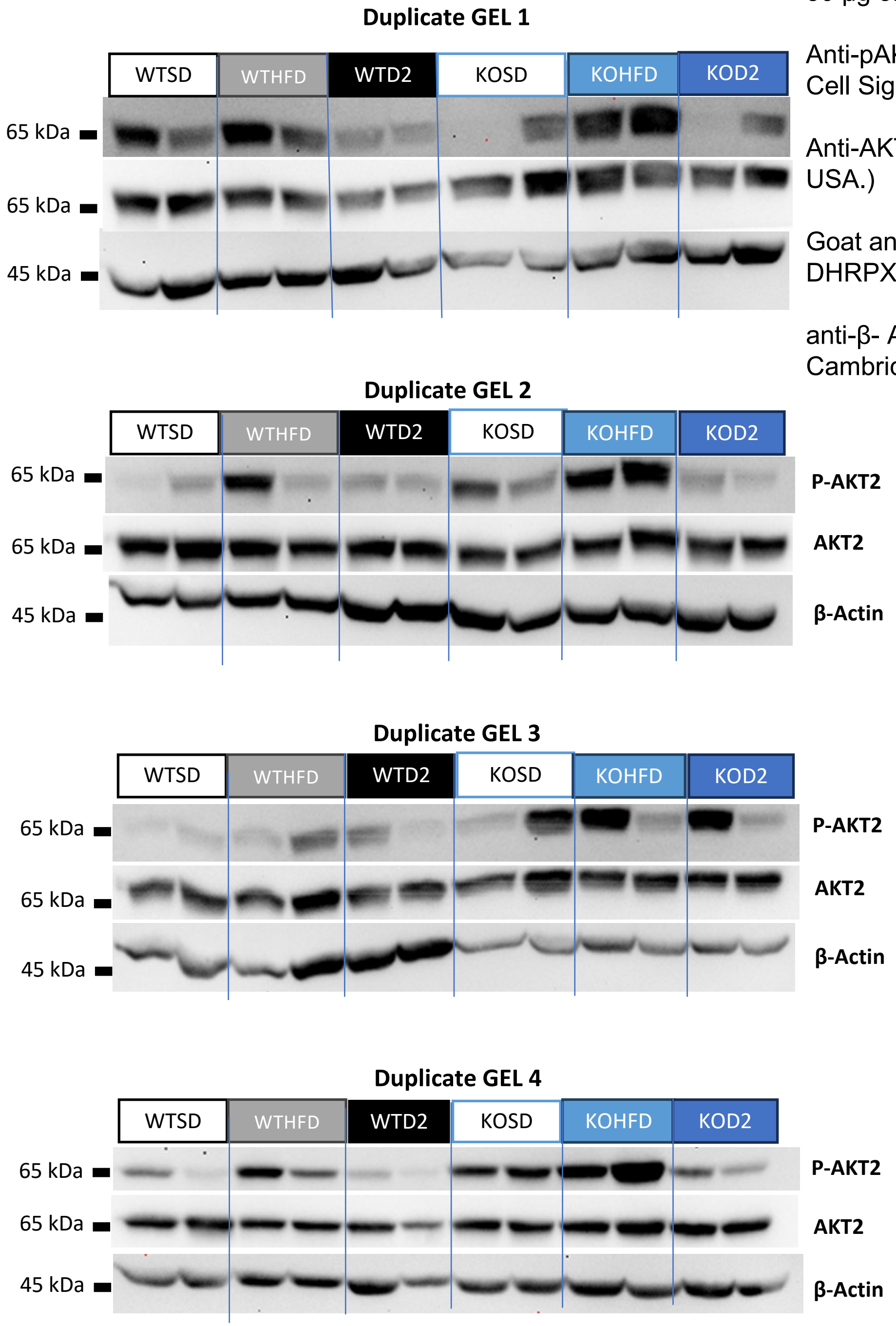


50 µg of liver tissue

Anti-pAKT2 (S474) 1: 2000 (Cat. No 8599S, Cell Signaling, MA, USA.)

Anti-AKT2 1: 2000 (Cat. No. 3063S, Cell Signaling, MA, USA.)

Goat anti-Rabbit IgG 1:5000 (Cat. No. GtxRb-003-DHRPX, ImmunoReagents, NC. USA.)

anti-β- Actin HRP 1:500,000 (Cat. No. ab49900, Abcam, Cambridge, UK.)

# SerpinA3k in the ADIPOSE TISSUE

5 µg of adipose tissue

Anti-SerpinA3k 1:1000, (Cat. No. 55480-1-AP, Proteintech, IL. USA.)
Goat anti-Rabbit IgG 1:5000 (Cat. NO. GtxRb-003-DHRPX, ImmunoReagents, NC. USA.)

**GEL 1**

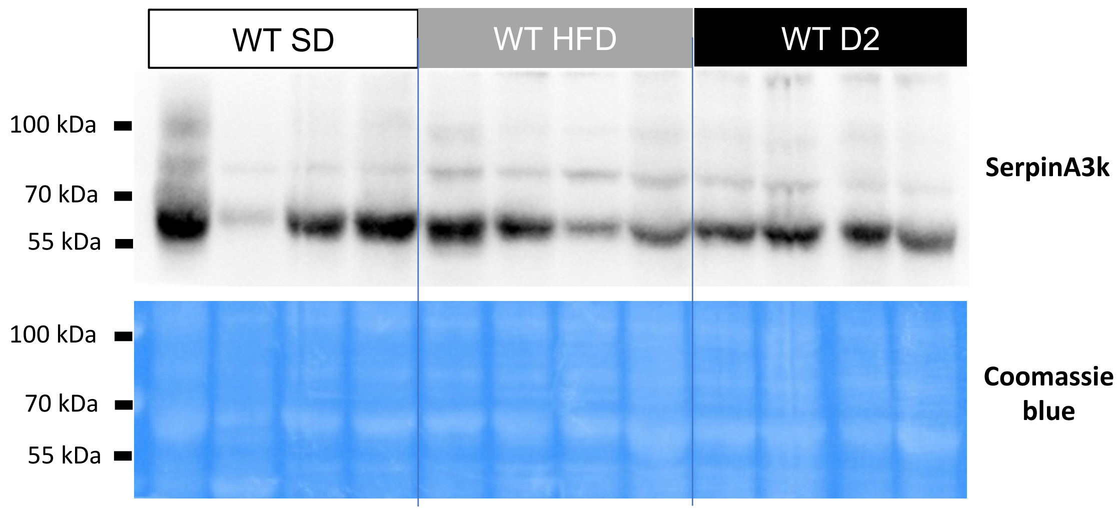


**Duplicate GEL 1**

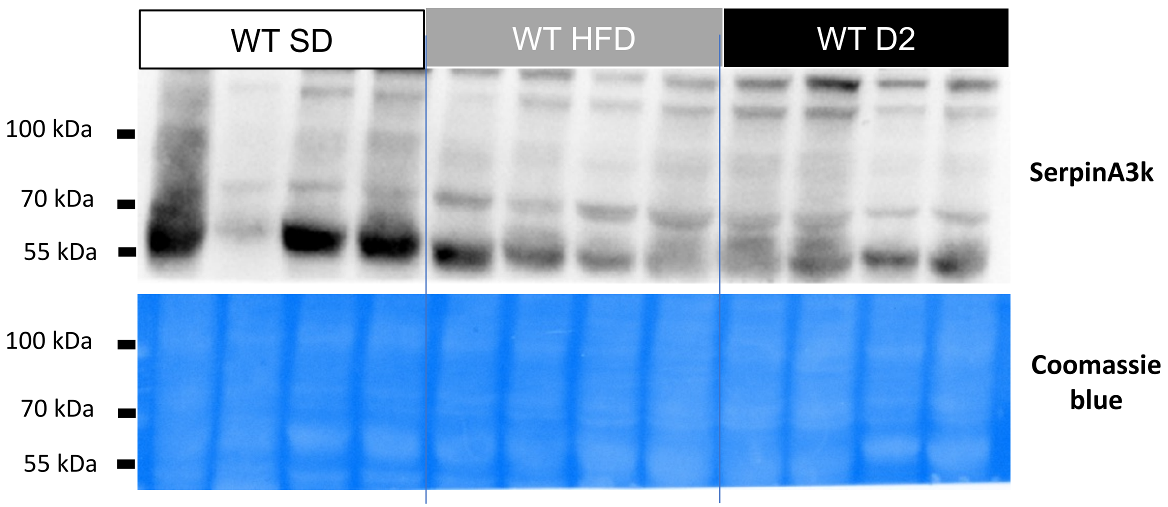


**GEL 2**

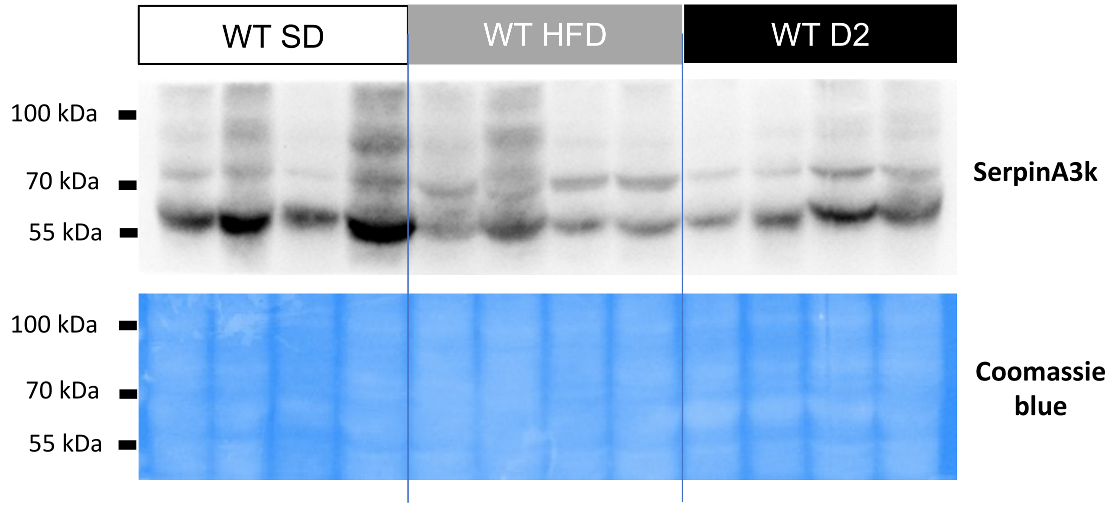


**Duplicate GEL 2**

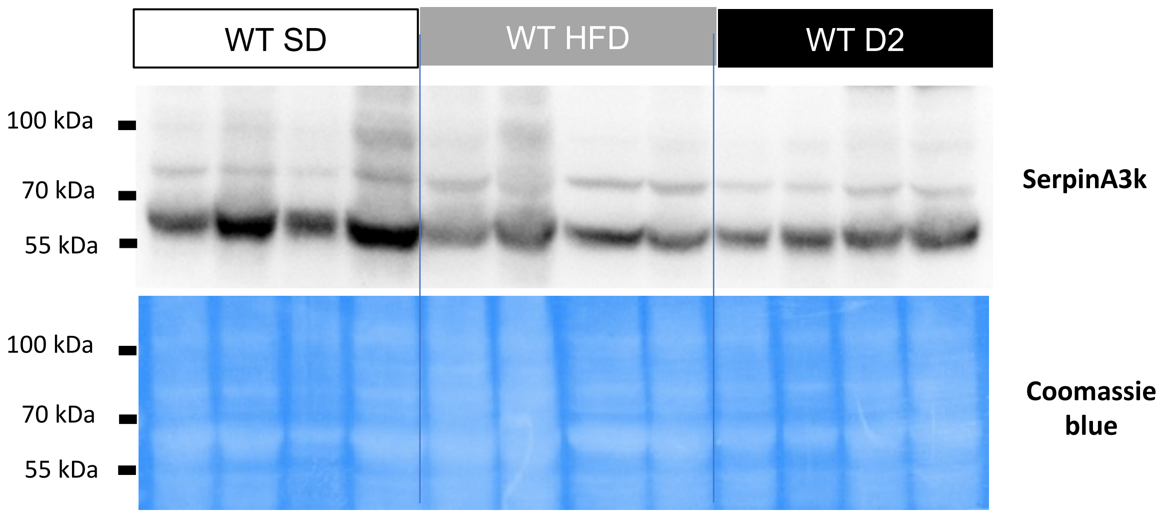

# URINARY SerpinA3k

10 µL of urine

Anti-SerpinA3k, 1:1000 (Cat. No. 55480-1-AP, Proteintech, IL. USA.)

Anti-rabbit 1:15,000 (Cat. No. 211-032-171, Jackson ImmunoResearch, PA., USA.)

**GEL 1**

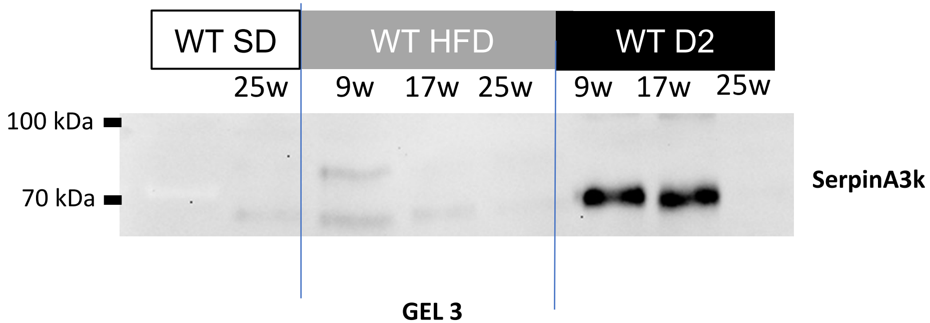


**GEL 2**

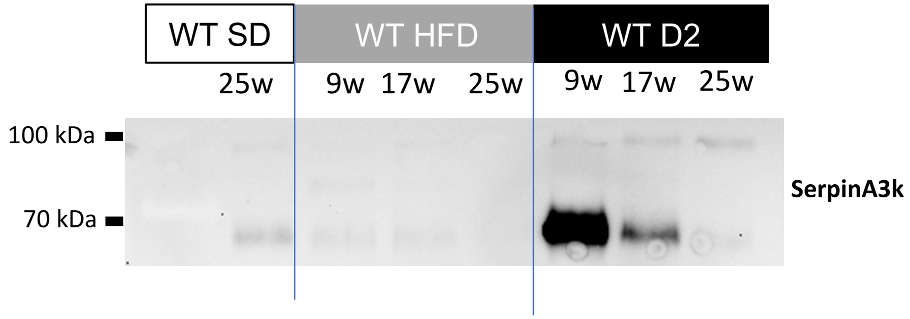


**GEL 3**

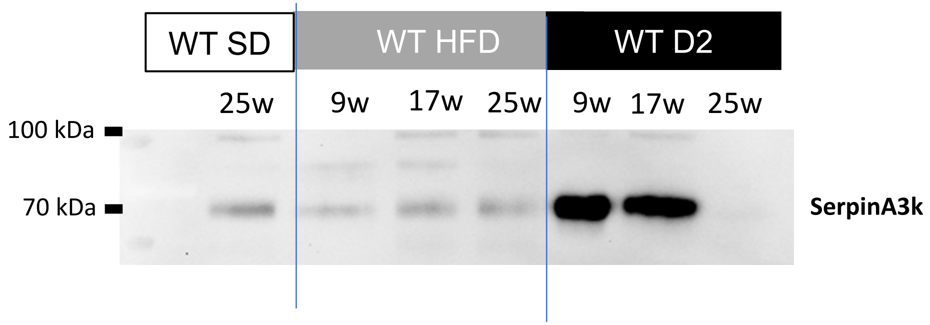


**GEL 4**

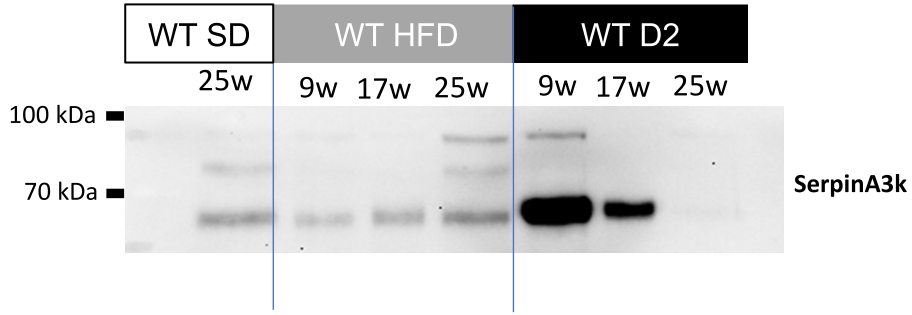


**GEL 5**

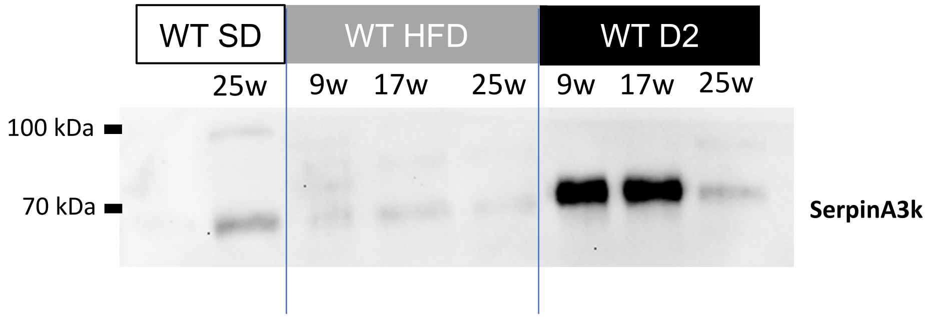


**GEL 6**

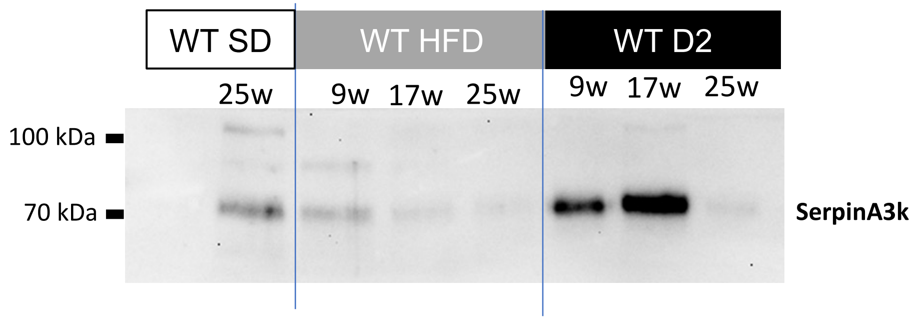

# SerpinA3k in the KIDNEY

25 µg of kidney tissue

Anti-SerpinA3k, 1:1000 (Cat. No. 55480-1-AP, Proteintech, IL. USA.)

Goat anti-Rabbit IgG, 1:5000 (Cat. No. GtxRb-003-DHRPX, ImmunoReagents, NC. USA.)

anti-β- Actin HRP 1:500,000 (Cat. No. ab49900, Abcam, Cambridge, UK.)

GEL 1

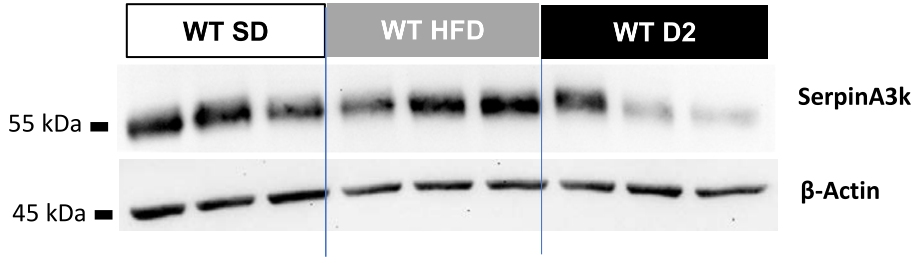


Duplicate GEL 1

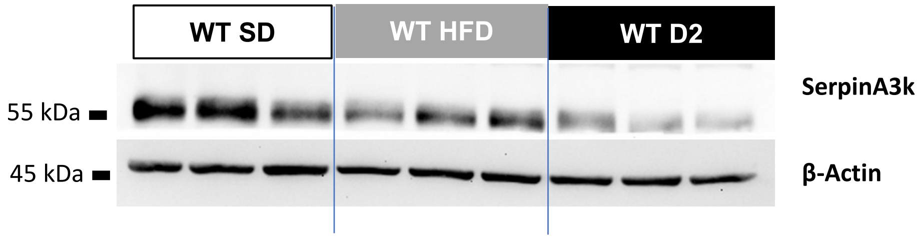


GEL 2

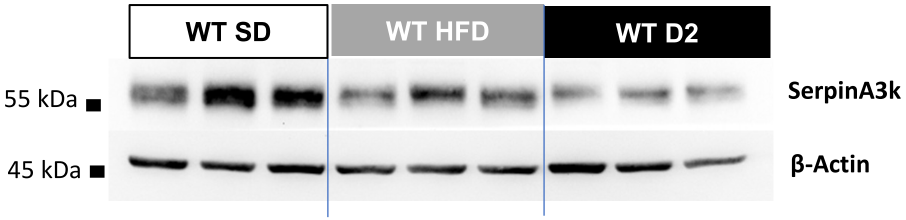


Duplicate GEL 2

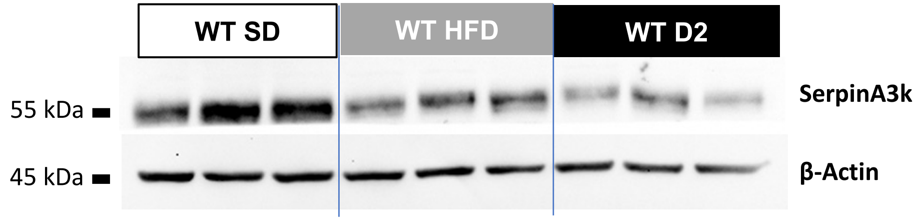

# IL-6 in the KIDNEY

25 µg of kidney tissue

Primary rabbit Anti-IL-6 (Cat. No. SABS700632, Sigma, USA) 1:1000

Goat anti-Rabbit IgG (Cat. No. A-0545, USA.) 1:10000

HRP anti-β- Actin (Cat. No-ab49900, Abcam, Cambridge, UK.) 1:500,000

GEL 1

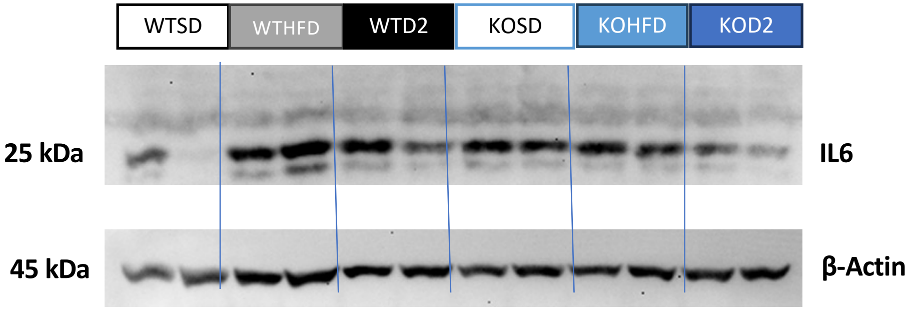


GEL 2

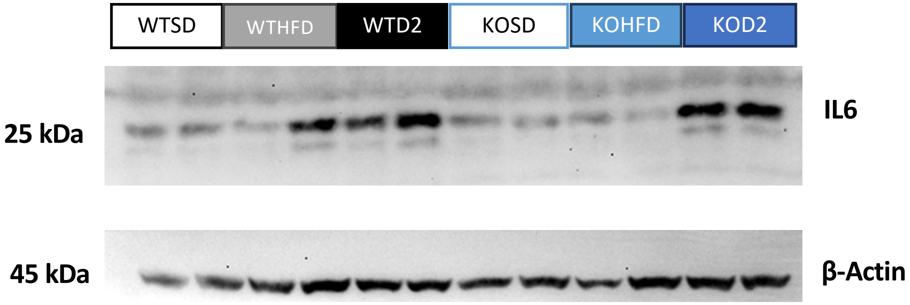


GEL 3

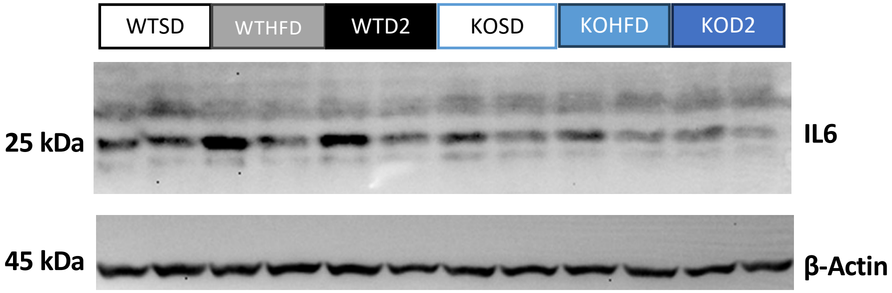


GEL 4

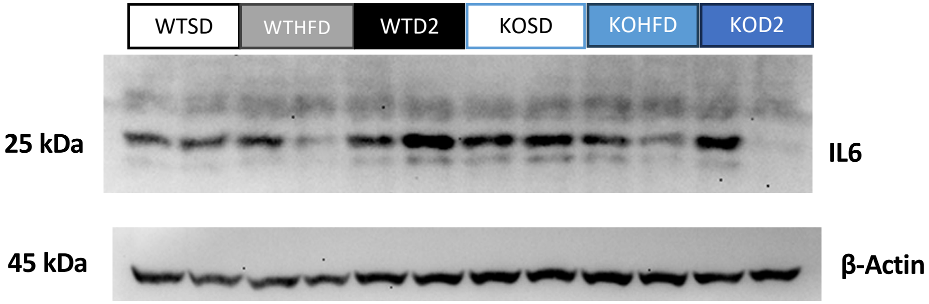

# TNFα in the KIDNEY

45 µg of kidney tissue

Primary rabbit Anti-TNFa (1:1,000; Cell Signaling, Cat. No. 3707), 1:1000

Goat anti-Rabbit IgG (Cat. No. A-0545, USA.) 1:10000

HRP anti-β- Actin (Cat. No-ab49900, Abcam, Cambridge, UK.) 1:500,000

GEL 1

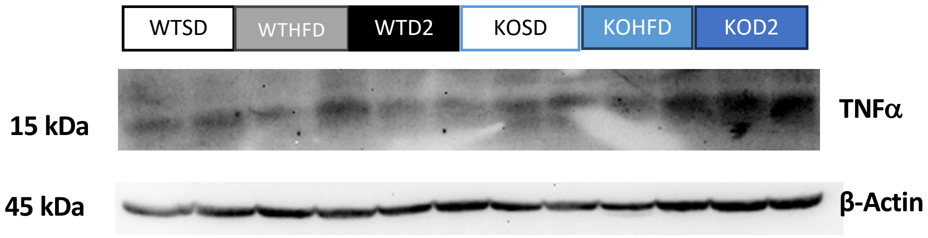


GEL 3

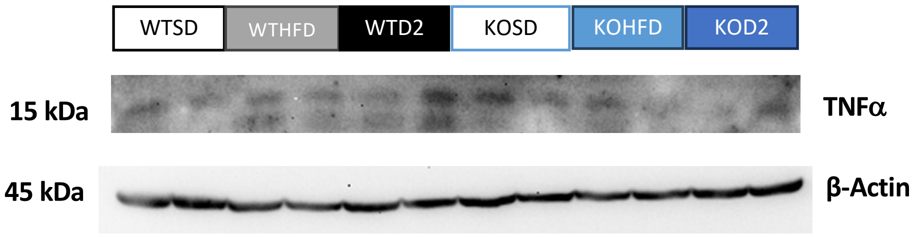


GEL 2

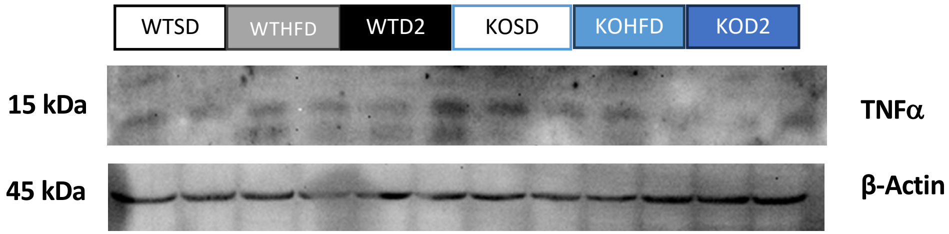


GEL 4

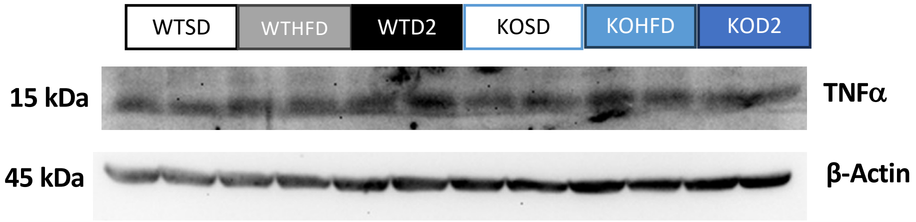

Supplement: Supplementary file 3 — Supplementary Material 3 (PDF 4.57 MB) [file 18_2025_5922_MOESM3_ESM.pdf]
